# Immunoadsorption and subsequent immunoglobulin G replacement (IA/IG) in patients with dilated cardiomyopathy: a systematic review and meta-analysis

**DOI:** 10.3389/fcvm.2026.1840280

**Published:** 2026-06-23

**Authors:** Xiao Xia, Yuhao Yao, Jun Li, Shiyi Tao, Xuanchun Huang, Chao Meng, Yiying Liu, Yonghao Li, Ruikang Liu

**Affiliations:** 1 Guang’anmen Hospital, China Academy of Chinese Medical Sciences, Beijing, China; 2 Graduate School of China Academy of Chinese Medical Sciences, Beijing, China; 3 Beijing University of Chinese Medicine, Beijing, China

**Keywords:** dilated cardiomyopathy, heart failure, immunoadsorption, meta-analysis, systematic review

## Abstract

**Background:**

Dilated cardiomyopathy (DCM) is a refractory cardiac disease with significant morbidity and mortality. Although immune adsorption combined with immunoglobulin G replacement therapy (IA/IG) has shown potential in treating DCM, only small-scale clinical trials have been reported. Its efficacy and safety characteristics still need to be further systematically evaluated.

**Methods:**

We conducted a systematic review and meta-analysis in accordance with PRISMA 2020 guidelines. A comprehensive search of PubMed, Embase, Web of Science, and the Cochrane Library was performed up to July 1, 2025. Clinical studies on IA/IG treatment for DCM were included. The primary outcome was the left ventricular ejection fraction (LVEF) change. Secondary outcomes included left ventricular end-diastolic dimension (LVEDD), NYHA functional class, N-terminal pro-brain natriuretic peptide (NT-proBNP), and VO2 peak. Study quality and risk of bias were assessed using the Cochrane ROB 2.0 and ROBINS-I tools. Sensitivity analysis was taken into consideration to determine the stability of the results. This review was registered with PROSPERO (CRD420251104796).

**Results:**

Eighteen studies (2 RCTs and 16 non-RCTs) involving 809 participants were included. IA/IG therapy significantly improved LVEF [mean difference (MD) = 7.71%, 95% CI: 6.18–9.24, *p* < 0.00001] and reduced LVEDD (MD = −3.22 mm, 95% CI: −4.16 to −2.28, *p* < 0.00001) from baseline. Significant improvements were also observed in NYHA functional class (MD = −0.76, 95% CI: −0.91 to −0.60, *p* < 0.00001) and peak VO₂ (MD = 2.66 mL/kg/min, 95% CI: 1.26–4.06, *p* = 0.0002). Compared to controls, the IA/IG group demonstrated greater improvement in LVEF (MD = 8.31%, 95% CI: 6.45–10.18, *p* < 0.00001) and NYHA class (MD = –0.62, 95% CI: −1.00 to −0.25, *p* = 0.001). A sensitivity analysis of the results suggested that they were stable.

**Conclusion:**

Systematic reviews and meta-analyses suggest that IA/IG therapy may improve cardiac function and quality of life in patients with DCM. However, the number of RCTs included in the study is limited, so these results should be interpreted with caution. Further high-quality, large-scale trials are warranted to establish standardized treatment protocols and confirm the long-term benefits of IA/IG therapy.

**Systematic Review Registration:**

https://www.crd.york.ac.uk/PROSPERO/recorddashboard, PROSPERO CRD420251104796.

## Introduction

Dilated cardiomyopathy (DCM) is a primary heterogeneous cardiomyopathy characterized by significant dilatation and systolic dysfunction of the left ventricular or biventricular chambers, with a core pathophysiological manifestation of diminished myocardial contractility and reduced ejection fraction, accompanied by thinning of the ventricular wall or compensatory hypertrophy, ultimately leading to congestive heart failure, arrhythmia, or sudden death (1–3). According to epidemiological research, the global prevalence of DCM is estimated at 3.2 million cases and is an essential cause of sudden cardiac death in young and middle-aged adults (4). In recent years, the incidence of DCM has gradually increased and even tends to be younger. The 5-year morbidity and mortality rate of DCM in Europe is reported to be about 15%–50%, and in the United States, the annual death rate of DCM is about 3.23/100,000 people. In China, there are 130,000–84,000 new patients with DCM each year, with a 5-year mortality rate of about 42.24% and a 10-year survival rate of less than 25% (5). Moreover, death can occur at any stage of the disease, and the prognosis is inferior, which makes DCM a clinically recognized refractory cardiomyopathy. Currently, the pathogenesis of DCM is unclear and is usually thought to be related to genetic factors, autoimmune diseases, viral infections, and chemotherapeutic agents (1). DCM management aims to reduce heart failure symptoms and improve cardiac function, prevent sudden death and embolism, and improve patient quality of life and survival (6). With the clinical application of novel drugs such as angiotensin receptor-enkephalinase inhibitors (ARNI), sodium-glucose co-transporter protein 2 inhibitors (SGLT-2), and sGC stimulators, the rate of heart failure hospitalization and the risk of the composite endpoint of cardiovascular death in patients with DCM can be significantly reduced (7, 8). However, most patients with end-stage DCM have unsatisfactory pharmacologic outcomes, and heart transplantation remains the only therapeutic option. The shortage of donors, clinical contraindications, and financial burden, among other factors, prevent the majority of DCM patients from receiving heart transplantation. Therefore, immunosorbent therapy regimens may be a promising complementary treatment option.

Immunoadsorption (IA) combined with immunoglobulin replacement (IG) therapy (IA/IG) is a targeted strategy for autoantibody-mediated myocardial injury. Immunoabsorption improves cardiac function in the short term by specifically removing pathogenic autoantibodies from the patient's blood, followed by supplementation with intravenous immunoglobulin (IVIG) to neutralize residual antibodies and regulate immune homeostasis (9). The combination regimen (IA followed by IG) synergizes rapid clearance with long-term protection. Most of the present research on IA/IG is focused on the treatment of autoimmune diseases, such as systemic lupus erythematosus and Guillain-Barrésyndrome (10, 11). However, it has been preliminarily shown that IA/IG can improve cardiac function and morphologic parameters, alleviate clinical symptoms, decrease clinical biomarkers associated with heart failure, increase exercise tolerance, and improve endothelial function in DCM patients (12–15). However, these trials were reported in small numbers, included fewer patients, and were inconsistently designed. Thus, further systematic evaluation is required.

This study aims to comprehensively assess the efficacy and safety of IA/IG therapy in the treatment of DCM by systematically evaluating existing clinical trial data. It will, in turn, provide an important reference for the subsequent development of more accurate clinical trial design and individualized treatment plan development, thus promoting the development of clinical treatment for DCM.

## Methods

This study was reported following the Preferred Reporting Items for Systematic Reviews and Meta-Analyses (PRISMA 2020) statement (Supplementary File S1). The review program was registered with the International Prospective Register of Systematic Reviews (PROSPERO) under the registration number CRD420251104796.

### Search strategy

Two researchers independently searched PubMed, Embase, Web of Science Core Collection, and Cochrane Library databases for the terms “Dilated cardiomyopathy”, “Immunosorption”, “IA/IG”, “Immunoglobulin”, and “Immunotherapy” with a timeframe of construction to July 1, 2025 (Supplementary File S2). Search strategies were tailored for each database, and further relevant studies were manually retrieved from the databases.

### Study selection

Studies were considered to meet the inclusion criteria if they met the following criteria：

1. Participants: Patients enrolled in the study were diagnosed with DCM based on the diagnostic criteria (16). We imposed no limitations on gender, nationality, or ethnic origin.
2. Intervention and control: Patients in the experimental group received IA/IG therapy along with the optimal medical treatments for DCM, while the control group received optimal medical treatments alone. There were no restrictions on duration or column type.
3. Outcome: The primary outcome was left ventricular ejection fraction (LVEF), and the secondary outcomes included left ventricular end-diastolic dimension (LVEDD), NYHA functional classification, N-terminal probrain natriuretic peptide (NT-proBNP), and VO2 Peak.
4. Study design: The study was an observational study (cohort or case-control) or a randomized trial with no limitations on language, publication year, publication status, or blinding methods.

The exclusion criteria are as follows:

1. Conference abstracts, reviews, comments, opinions, letters, and editorials.
2. Case reports, biochemical and experimental studies.
3. Incomplete or imprecise data or unavailable studies of the entire text.

### Data extraction

The literature from multiple databases was organized using EndNote X9 software. Two researchers independently screened the literature according to the inclusion criteria. After identifying duplicate studies, the researchers excluded irrelevant material by reviewing titles and abstracts. Finally, the entire text was reviewed again to identify eligible studies. Two researchers individually extracted data from the eligible studies according to a specially designed form. Included items included the first author, year of publication, country, type of study design, sample size, age, baseline LVEF level, baseline cardiac function classification, type of immunosorbent column, follow-up, outcome metrics, and evaluation of literature quality. A third investigator resolved any disagreements.

### Quality assessment

The RCT was independently quality assessed by 2 researchers using the ROB 2.0 tool (17) published on the official Cochrane website. The evaluation consisted of 5 modules: (1) bias arising from the randomization process, (2) bias due to deviations from intended interventions, (3) bias due to missing outcome data, (4) bias in measurement of the outcome, (5) bias in selection of the reported result. The risk of bias in each module was rated as “low risk,” “some concerns,” or “high risk,” and the overall risk of the literature was judged by the researcher based on the results of the five modules and the actual situation.

The non-RCT was assessed using the ROBINS-I V2 (18) quality assessment tool. Risk of bias included seven dimensions: (1) bias due to confounding, (2) bias in selection of participants into the study, (3) bias in classification of interventions, (4) bias due to deviations from intended interventions, (5) bias due to missing data, (6) bias in measurement of outcomes, (7) bias in selection of the reported result. The study's risk of bias before, during, and after the intervention was thoroughly evaluated by answering 33 landmark questions in these 7 bias modules. The third researcher was consulted, and any existing differences were discussed.

### Statistical analysis

Review Manager 5.4 for Meta-analysis. Meta-analysis was performed if two or more studies provided the same results. For continuous variables, in RCTs and case-control studies, the mean difference (MD) and 95% confidence interval (CI) between the two groups were determined, and the fraction of change from the baseline value was summarized. For comparisons between the experimental and control groups, the MD was calculated based on the change from baseline to follow-up (follow-up value minus baseline value) within each group; thus, the MD in the forest plots represents the “difference in change from baseline” between the two groups, rather than the difference in absolute post-intervention values. In cohort studies, the post-intervention change from baseline was compared. For studies with quantitative data that cannot be statistically synthesized, narrative synthesis of quantitative data is often the method of choice. Narrative synthesis is the organization of research findings into a coherent textual narrative that describes the differences in the research characteristics. For dichotomous data, CI and risk ratio were calculated. Heterogeneity between effects was assessed using the Cochrane Q test and the I^2^ statistic. According to the Cochrane Handbook (19), I^2^ ≤ 50% was considered to be a low to moderate heterogeneity, while I^2^ ≥ 75% indicated high heterogeneity. Fixed-effects models were used for homogeneous data, while random-effects models were used to analyze heterogeneous data. Funnel plots were used for graphical assessment of publication bias, and Egger's test was used to test for asymmetry in the funnel plots. Sensitivity analysis was performed by excluding highly biased studies. All *P* values were two-tailed and regarded as significant when *P* < 0.05.

## Results

**Figure 1:**
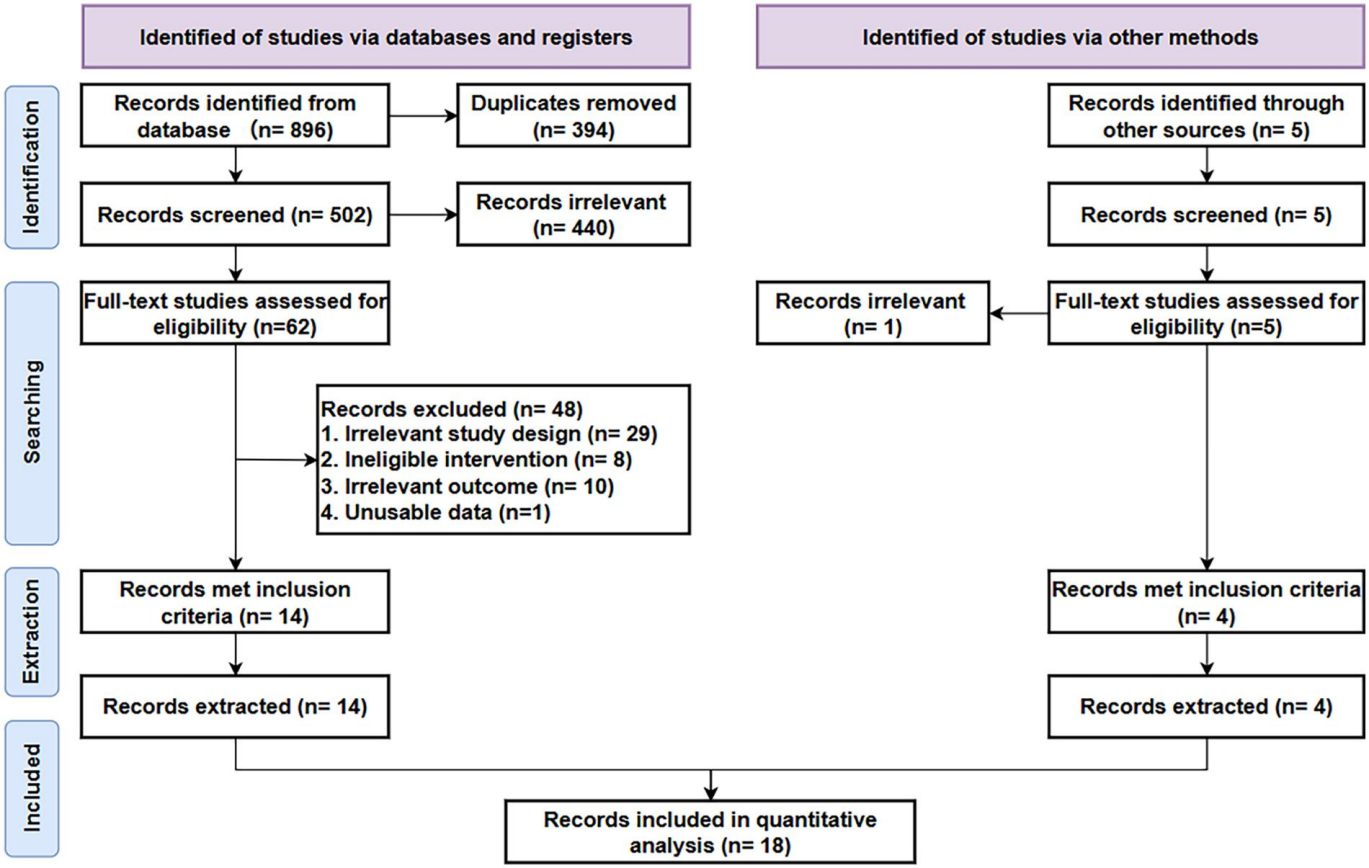
Flow diagram of study selection.

An initial search of 896 records was conducted from the database. After screening, 62 studies were eligible and passed checking, of which 48 were further excluded due to (1) unrelated study design (*n* = 29), (2) ineligible interventions (*n* = 8), (3) unrelated outcomes (*n* = 10), and (4) unavailable data for which study authors could not be contacted to provide summary statistics (*n* = 1). In addition, 5 studies were obtained from other sources, and 1 study was excluded due to irrelevant reports. After screening, 18 studies were finally included (Figure 1).

### Study characteristics

A total of 16 non-RCTs and 2 RCTs with 809 participants were included. The 2 RCTs included 21 people in the experimental group and 22 in the control group. Three of these studies categorized patients into responders and non-responders based on the degree of improvement in LVEF after receiving IA/IG. Also, outcome indicators were reported separately for both groups of patients. In our study, both groups of patients were included in the analysis and were differentiated by “a” and “b” after the author and year. (“a” is for responders and “b” is for non-responders). In all included studies, the mean age of the patients was greater than 40 years, and the baseline LVEF was less than 40%. Of the 18 trials, only one was in Turkey, and the rest were from Germany. The included trials were conducted from 2000 to 2022. All participants received conventional medication, including diuretics, ACEI/ARB, ARNI, hydrocorticoid receptor antagonists, cardiac glycosides, nitrates, and beta-blockers. The experimental group received IA/IG therapy based on medication. Protein A columns were the most frequently used immunosorbent columns. Follow-up time was 3–6 months in most studies. The characteristics of the included studies are shown in Table 1.

**Table 1:** Characteristics of the included studies.

No	Study ID	Country	Study design	Patients		Age (years)		Baseline LVEF (%)		NYHA	Type of columns	Follow-up	Outcomes
				E	C	E	C	E	C				
1	Herda 2010 (23)	Germany	PC	30	30	54.7 ± 1.9	52.6 ± 2.5	33.0 ± 1.2	30.1 ± 1.2	Ⅱ-Ⅳ	protein A	3 mo	①②⑤
2	Staudt 2006 (24)	Germany	PC	15	15	50.2 ± 2.0	52.4 ± 2.0	29.7 ± 1.0	28.1 ± 1.0	Ⅱ-Ⅳ	protein A	3 mo	①②④
3	Staudt 2001 (20)	Germany	PC	12	13	50.1 ± 3.3	49.8 ± 3.4	21.3 ± 1.7	18.3 ± 1.7	Ⅱ-Ⅳ	NR	3 mo	①②
4	Knebel 2004 (29)	Germany	RC	17	17	55	57	19.8 ± 6.4	24.6 ± 5.5	Ⅱ-Ⅳ	NR	2.5 y	①③
5	Kallwellis 2007 (30)	Germany	RC	6	6	41.5 ± 6.3	45.3 ± 4.2	27.7 ± 2.7	32.2 ± 1.4	Ⅰ-Ⅲ	NR	3 mo	①
6	Felix 2000 (21)	Germany	PC	9	9	49.7 ± 3.2	55.3 ± 2.4	20.1 ± 1.2	22.6 ± 1.7	Ⅱ-Ⅳ	NR	3 mo	①③
7	Voigt 2010 (25)	Germany	PC	90		51.1 ± 0.9		32.6 ± 0.7		Ⅱ-Ⅳ	protein A	6 mo	①
8	Trimpert 2010a (31)	Germany	PC	11		52.8 ± 3.4		33.8 ± 1.7		Ⅱ-Ⅳ	protein A	12 mo	①②
	Trimpert 2010b (31)			6		52.8 ± 2.5		32.0 ± 4.0					
9	Staudt 2010 (26)	Germany	PC	103		50.7 ± 1.0		32.2 ± 0.7		Ⅱ-Ⅳ	NR	6 mo	①②③
10	Reinthaler 2014 (27)	Germany	RC	15		58.4 ± 9.3		33.0 ± 6.4		Ⅰ-Ⅲ	protein A	3–6 mo	①②③⑤
11	Ohlow 2016 (32)	Germany	PC	93		61.0 ± 11.1		30.0 ± 11.1		Ⅱ-Ⅳ	protein A	12 mo	①②③④
12	Doesch 2010 (33)	Germany	PC	51		53.1 ± 12.4		26.3 ± 9.4		Ⅱ-Ⅳ	protein A	6 mo	①④⑤
13	Dandel 2012 (22)	Germany	RC	116		47.6 ± 11.5		23.6 ± 5.1		Ⅱ-Ⅳ	polyclonal anti-human antibodies、synthetic peptide	63–176 mo	①②③④⑤
14	Dandel 2015 (14)	Germany	PC	62		47.8 ± 10.3		23.8 ± 6.8		Ⅰ-Ⅳ	synthetic peptide	1–10 y	①②④⑤
15	Cavusoglu 2022 (34)	Turkey	PC	9		44.1 ± 7.8		23.3 ± 5.1		Ⅲ-Ⅳ	tryptophan	6 mo	①②④
16	Bulut 2012 (35)	Germany	PC	18		50.9 ± 8.8		27.6 ± 6.4		Ⅱ-Ⅳ	protein A	6 mo	①②③④⑤
17	Bhardwaj 2016a (28)	Germany	RC	7		44.0 ± 13.0		33.0 ± 8.4		Ⅱ-Ⅲ	protein A	6 mo	①②
	Bhardwaj 2016b (28)			9		52.0 ± 9.0		34.0 ± 8.0					
18	Ameling 2012a (36)	Germany	PC	24		49.0 ± 10.0		33.0 ± 6.0		Ⅱ-Ⅲ	protein A	6 mo	①②③
	Ameling 2012b (36)			16		52.0 ± 9.0		34.0 ± 7.0					

E, experimental group; C, control group; NR, not reported; PC, prospective cohort; RC, retrospective cohort; a, IA/IG responders; b, IA/IG non-responders. ①, left ventricular ejection fraction (LVEF); ②, left ventricular end-diastolic dimension (LVEDD); ③, New York Heart Association (NYHA) functional classification; ④, N-terminal pro-brain natriuretic peptide (NT-proBNP); ⑤, VO2 peak. Higher LVEF and VO2 peak values indicate a better outcome, and lower values of LVEDD, NYHA functional classification, and NT-proBNP indicate a better outcome.

### Quality evaluation

**Figure 2:**
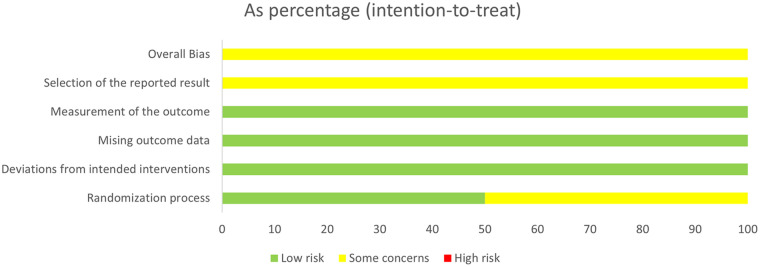
Risk-of-bias graph of RCTs.

The ROB 2.0 tool was used to evaluate the quality of the included RCTs. Only 2 of the included studies were RCTs (20, 21). One study (20) mentioned “closed label” during randomization and was rated as a “low risk” of bias. The other study (21) only mentioned randomized groups and was rated as “some concerns”. Neither study provided more information on allocation concealment and blinding. Measurement of outcome indicators was rated as “low risk” because in both studies, the outcome indicators were performed by blinded assessors. Regarding the selective reporting of outcome indicators, all included literature was rated as “some concerns” risk for not blinding the researchers. During the intervention, all the literature did not show any deviation from the established intervention, and no literature had missing data and was rated as “low risk”. The risk-of-bias graph of the included RCTs is presented in Figure 2.

The ROBINS-I V2 tool was used to evaluate the quality of the included non-RCTs. None of the included literature reported further management of confounders present at baseline. All literature was without subject selection bias and intervention categorization bias, and did not show deviation from established interventions. In terms of missing data, one study (22) did not adequately report data on dropout patients. In terms of outcome indicator measurement, six studies (23–28) mentioned assessment by a blinded examiner, and the rest did not. Regarding the selective reporting of outcome indicators, all included literature was rated as moderate risk for not blinding the investigator. The risk of bias evaluation for the inclusion of non-RCTs is shown in Table 2. Specific assessment entries are provided in the Supplementary File S3.

**Table 2:** Risk-of-bias assessment of non-RCTs.

Study ID	Bias due to confounding	Bias in the selection of participants into the study	Bias in the classification of interventions	Bias due to deviations from intended interventions	Bias due to missing data	Bias in the measurement of outcomes	Bias in the selection of the reported result	Overall bias
Bhardwaj 2016 (28)	Moderate	Low	Low	Low	Low	Low	Moderate	Moderate
Bulut 2012 (35)	Moderate	Low	Low	Low	Low	Moderate	Moderate	Moderate
Cavusoglu 2022 (34)	Moderate	Low	Low	Low	Low	Moderate	Moderate	Moderate
Dandel 2012 (22)	Moderate	Low	Low	Low	Moderate	Moderate	Moderate	Moderate
Dandel 2015 (14)	Moderate	Low	Low	Low	Low	Moderate	Moderate	Moderate
Doesch 2010 (33)	Moderate	Low	Low	Low	Low	Low	Moderate	Moderate
Herda 2010 (23)	Moderate	Low	Low	Low	Low	Low	Moderate	Moderate
Kallwellis 2007 (30)	Moderate	Low	Low	Low	Low	Moderate	Moderate	Moderate
Knebel 2004 (29)	Moderate	Low	Low	Low	Low	Moderate	Moderate	Moderate
Ohlow 2016 (32)	Moderate	Low	Low	Low	Low	Moderate	Moderate	Moderate
Reinthaler 2014 (27)	Moderate	Low	Low	Low	Low	Low	Moderate	Moderate
Staudt 2006 (24)	Moderate	Low	Low	Low	Low	Low	Moderate	Moderate
Staudt 2010 (26)	Moderate	Low	Low	Low	Low	Moderate	Moderate	Moderate
Trimpert 2010 (31)	Moderate	Low	Low	Low	Low	Moderate	Moderate	Moderate
Voigt 2010 (25)	Moderate	Low	Low	Low	Low	Low	Moderate	Moderate

### Outcomes

#### LVEF

**Figure 3:**
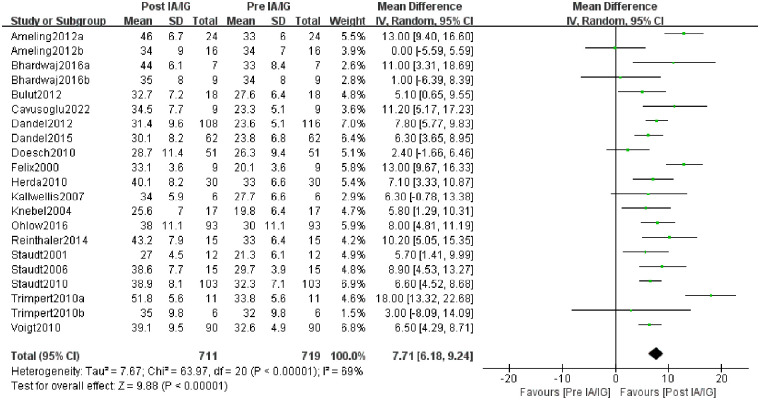
Forest plot of LVEF (comparison of IA/IG treatment before and after).

A total of 18 clinical studies (14, 23–36), including 801 participants, reported LVEF. The combined random effects model showed a statistically significant difference between before and after treatment with IA/IG (*MD* *=* *7.71, 95% CI 6.18∼ 9.24, P＜0.00001*). The heterogeneity test suggested a moderate degree of statistical heterogeneity (*I^2^* *=* *69%, P＜0.00001*) (Figure 3).

**Figure 4:**
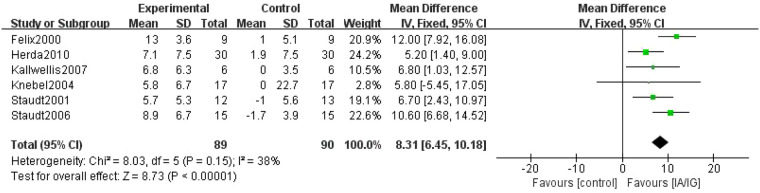
Forest plot of LVEF (comparison between the experimental group and the control group).

In addition, six trials (21–23), with 179 participants, provided information on LVEF in the experimental and control groups. Fixed-effects modeling was performed, and the results indicated a significant increase in mean LVEF (*MD* *=* *8.31, 95% CI 6.45∼10.18, P* *<* *0.00001*) in the experimental group. The heterogeneity test suggested low statistical heterogeneity (*I^2^* *=* *38%, P* *=* *0.15*) (Figure 4).

#### LVEDD

**Figure 5:**
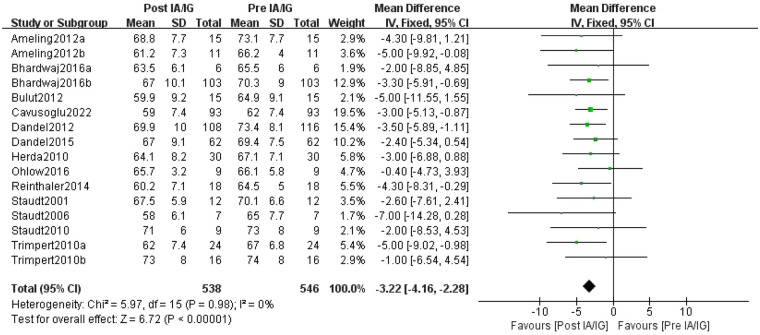
Forest plot of LVEDD (comparison of IA/IG treatment before and after).

13 studies (14, 20–36) with 596 participants provided LVEDD information before and after treatment with IA/IG. The combined results of the fixed-effects model showed a statistically significant difference between before and after treatment (*MD* *=* *−3.22, 95% CI −4.16∼−2.28, P＜0.00001*). The heterogeneity test suggested no statistical heterogeneity (*I^2^* *=* *0%, P* *=* *0.98*) (Figure 5).

**Figure 6:**
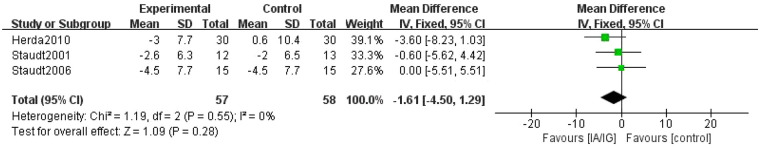
Forest plot of LVEDD (comparison between the experimental group and the control group).

Similarly, 3 studies (20–23) provided information on LVEDD in the experimental vs. control groups. The combined results of the fixed-effects model showed no statistically significant difference between the two groups (*MD* *=* *−1.61, 95% CI −4.50∼1.29, P* *=* *0.28*). The heterogeneity test suggested no statistical heterogeneity (*I^2^* *=* *0%, P* *=* *0.55*) (Figure 6).

### NYHA functional classification

**Figure 7:**
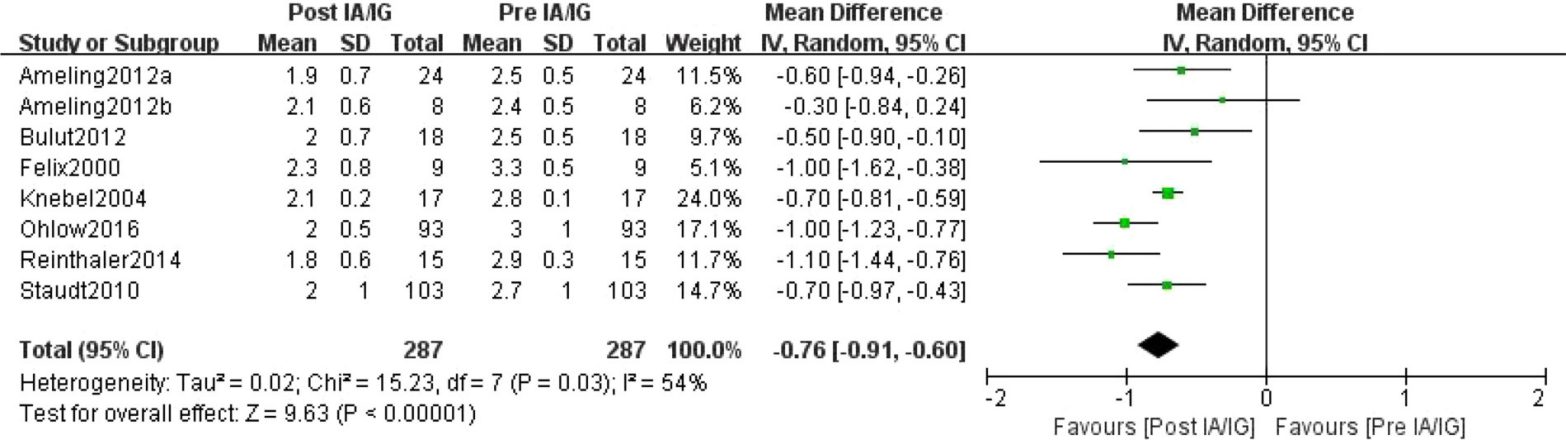
Forest plot of NYHA functional class (comparison of IA/IG treatment before and after).

7 clinical trials (21, 26–32, 35, 36) in 313 participants examined the effect of IA/IG treatment on the NYHA functional class. Random-effects modeling showed significantly better improvement in cardiac function after treatment than before (*MD* *=* *−0.761,95% CI −0.91∼−0.60, P* *<* *0.00001*). There was moderate heterogeneity in the included studies (*I^2^* *=* *54%, P* *=* *0.14*) (Figure 7).

**Figure 8:**
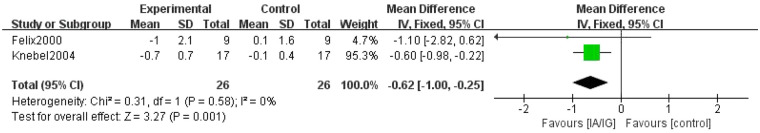
Forest plot of NYHA functional class (comparison between the experimental group and the control group).

Likewise, 2 studies (21, 29) with 52 participants reported differences in NYHA functional class between the experimental and control groups. Fixed-effects modeling showed that the difference in the combined means of the studies favored the experimental group and was statistically significant (*MD* *=* *−0.62,95% CI −1.00∼−0.25, P* *=* *0.001*). There was no heterogeneity in the combined analysis (*I^2^* *=* *0%, P* *=* *0.58*)(Figure 8).

### NT-proBNP

A total of 7 studies (14, 24, 32–35) reported NT-proBNP information for 371 participants. However, 5 of these studies (14, 32–34) were reported using quartiles because the data did not conform to normal distribution. The other 2 studies (24, 35) had significant differences in the way NT-proBNP was detected and the units of value. Data heterogeneity was high, so quantitative data analysis of NT-proBNP was not performed. 4 studies (24, 32–34) found a statistically significant difference in improvement of NT-proBNP after treatment with IA/IG compared with before treatment; 3 studies (14, 22, 35) found no statistically significant difference before and after treatment.

### VO2 peak

**Figure 9:**
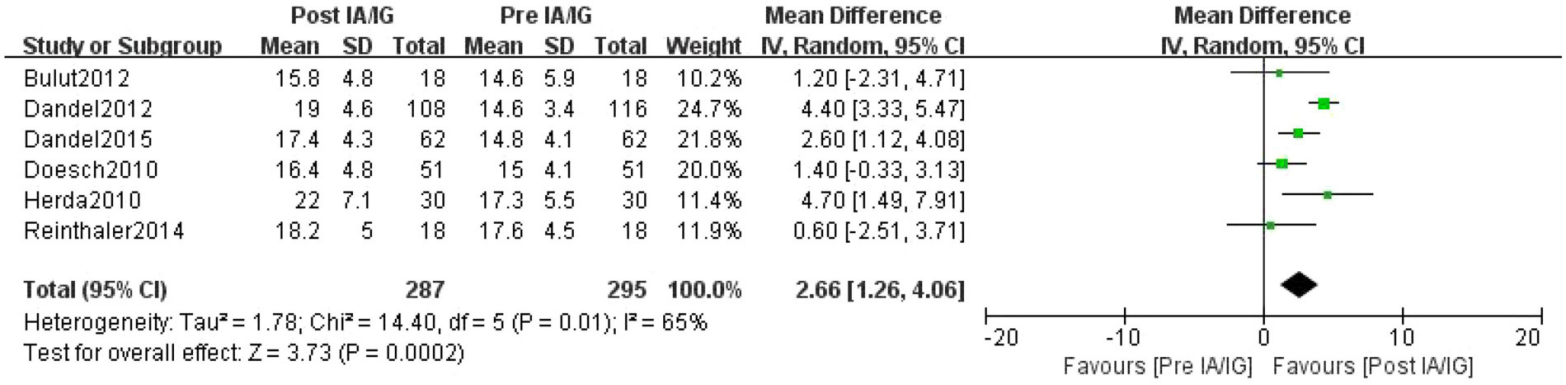
Forest plot of VO2 peak.

6 studies (14, 22, 23, 27, 33, 35) reported VO2 peak before and after IA/IG treatment in a total of 287 patients. Overall, the results of the random-effects model showed a significant difference in VO2 peak after IA/IG treatment compared with before treatment (*MD* *=* *2.66, 95% CI 1.26∼4.06, P* *=* *0.0002*) compared with before treatment. The heterogeneity test suggested a moderate degree of statistical heterogeneity (*I^2^* *=* *65%, P* *=* *0.01*))(Figure 9).

### Sensitivity analyses

Sensitivity analyses were performed separately for all results. The results indicated that each result was stable (Supplementary File S4).

### Publication bias

**Figure 10:**
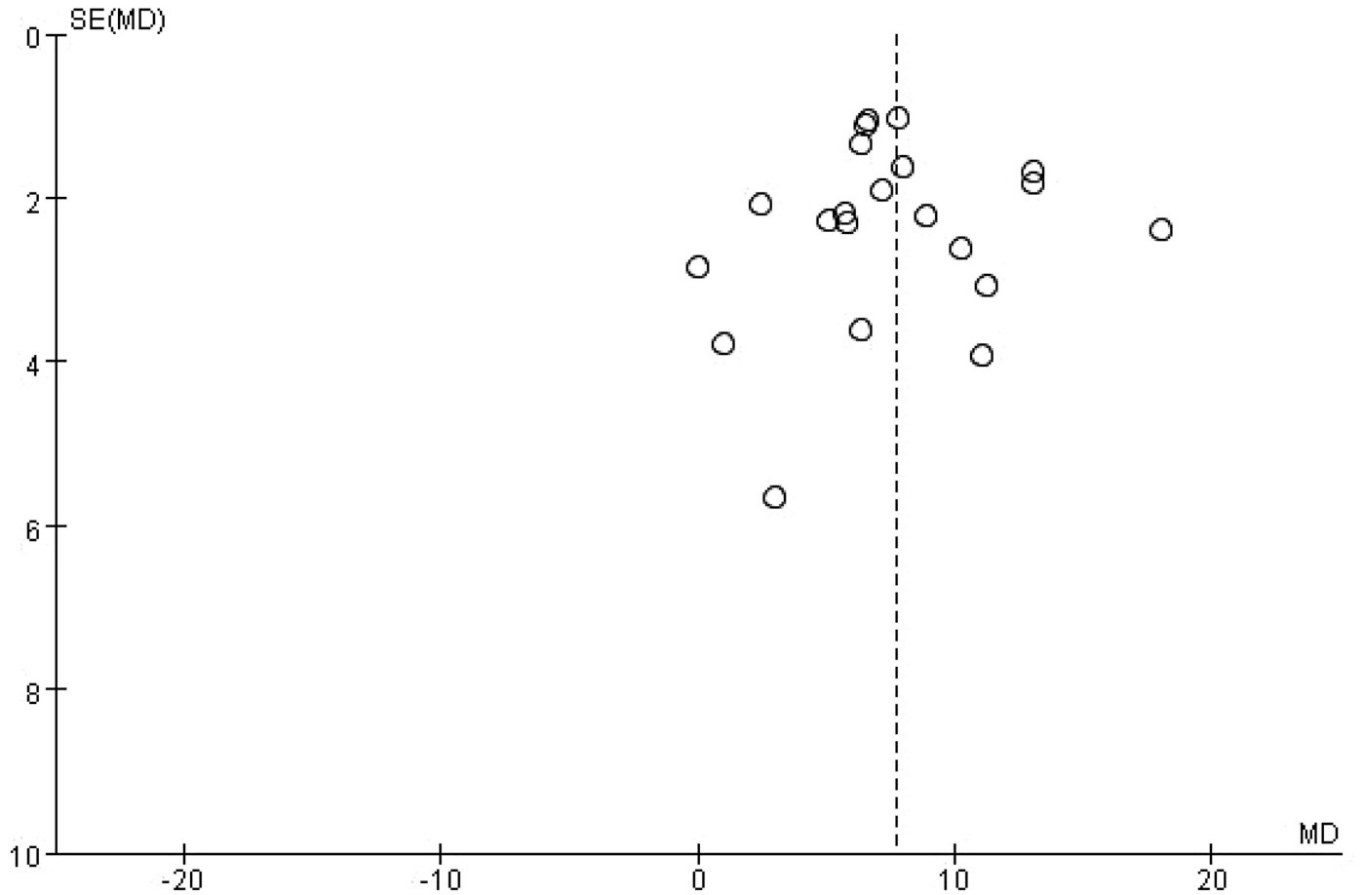
Funnel plot of included studies. Dots represent included studies, and the vertical line represents the pooled effect.

Funnel plots for all included studies showed an approximately symmetrical distribution of the point estimates around the effect size, indicating no signs of publication bias. Each point represents a study, and the blue dashed line represents the overall pooled effect. Egger’ s regression test for asymmetry confirmed this result, with a non-significant *p*-value (*P* *=* *0.103*)(Figure 10).

## Discussion

This systematic review and meta-analysis comprehensively evaluated the efficacy and safety of IA/IG in patients with DCM. Through the analysis of 18 clinical studies, we found that IA/IG treatment significantly improved patients’ cardiac function indicators, including LVEF, LVEDD, NYHA cardiac function classification, and VO₂ peak, with acceptable safety. Furthermore, sensitivity analysis showed that all results were stable. However, all studies included in the quality evaluation had a moderate risk of bias, so the significance of these findings should be cautiously addressed.

Our study found that IA/IG treatment increased LVEF by an average of 7.71%, which is similar to the 6.45% increase in LVEF reported in a previous systematic review and meta-analysis of IA treatment for DCM (37). The mechanisms by which IA/IG improve LVEF may involve the following aspects. The primary mechanism is the dual action of antibody clearance and immune regulation. IA can efficiently remove pathogenic autoantibodies (such as anti-*β*₁-adrenergic receptor antibodies and anti-M2-muscarinic receptor antibodies) from circulation through specific adsorption columns. A systematic review and meta-analysis found that DCM patients are more likely to have elevated levels of anti-*β*1-AR, M2-R, ANT, and myosin autoantibodies compared to healthy individuals. In particular, anti-*β*1-AR, calcium channel, and TnIAbs antibodies may play an important role in DCM's severity and poor prognosis (38). Additionally, increasing evidence has shown that the worsening of DCM-related heart failure is positively correlated with the prevalence of functional *β*1-adrenoreceptor autoantibodies (*β*1AR-AAbs) in patients (39–41). The activation of these antibodies can induce cytotoxicity, interfere with myocardial cell signaling, and ultimately lead to myocardial cell apoptosis and cardiac dysfunction (42, 43). The efficient removal of pathogenic antibodies by IA can improve left ventricular ejection fraction, increase left ventricular stroke volume, and cardiac output in the long term (44). Similarly, intravenous immunoglobulin (IVIG) can also specifically bind to and neutralize pathogenic autoantibodies remaining in the patient's body, blocking their continued damage to myocardial cells and thereby restoring myocardial contractile function. In addition, IVIG can also suppress B cell activation and autoantibody production, inhibit the release of proinflammatory cytokines (such as TNF-αand IL-6), alleviate myocardial inflammation, and improve the local immune microenvironment of the myocardium (45, 46). Furthermore, there are studies that suggest that the mechanism by which IA/IG improves cardiac function is also related to improving myocardial energy metabolism and reducing oxidative stress damage (34, 47).

Importantly, the interpretation of the therapeutic effect of IA/IG in DCM should not rely solely on the presence of autoantibodies, but also on whether these autoantibodies are functionally active. Not all detectable autoantibodies are pathogenic; only those capable of binding to and modulating the function of their target antigens directly contribute to myocardial injury and progressive cardiac dysfunction. This issue is particularly relevant for *β*1AR-AABs, which are extensively investigated in DCM due to their established association with the severity of heart failure. Functional *β*1AR-AABs, which can be identified by bioassays using spontaneously beating neonatal rat cardiomyocytes, are detectable in up to 97% of DCM patients with end-stage heart failure. Remarkably, IA therapy has been shown to effectively remove these functional antibodies in approximately 80% of cases, often reducing them below pathological levels (40, 48). Furthermore, Wallukat et al. further suggested that the removal of functional *β*1AR-AABs may contribute to delaying heart transplantation in selected patients (49)^.^ Despite these promising findings, there is currently no standardized method for the detection and quantification of functional AABs, which limits their routine clinical application. This lack of standardization may contribute to the observed variability in patient response to IA across studies, as the presence of functionally significant autoantibodies, rather than mere serological antibody positivity, is a key determinant of therapeutic efficacy. Future research should focus on developing practical, standardized, and clinically accessible methods for the assessment of functional AABs to enable more accurate patient stratification and enhance treatment outcomes.

Additionally, our analysis showed that patients who received IA/IG treatment had improved LVEDD compared to before treatment. Staudt et al. have demonstrated that the immunomodulatory effects of IA/IG therapy may improve ventricular remodeling by reducing myocardial inflammatory responses. IA/IG therapy reduces CD3+ T lymphocyte infiltration in the myocardium and decreases HLA class II antigen expression (20). This anti-inflammatory effect may interrupt the vicious cycle between immune-mediated myocardial damage and ventricular dilatation. Secondly, the improvement in LVEDD may be related to the restoration of calcium regulation and energy metabolism in cardiomyocytes (50, 51). It is worth noting that this study found that the improvement in LVEDD persisted during the 6-month follow-up period, suggesting that IA/IG therapy may have a lasting effect on ventricular remodeling. This finding is significant because progressive ventricular dilatation is a marker of DCM progression and is closely associated with poor clinical outcomes (52). However, it should be pointed out that there was no statistically significant difference between the experimental group and the control group in terms of LVEDD improvement. This may be due to sample size limitations and patient heterogeneity. Not all patients respond identically to IA/IG therapy in terms of LVEDD improvement. This variability may reflect differences in the underlying pathophysiological mechanisms of DCM or variations in individual antibody profiles among patients. Future studies are needed to further explore predictive markers for ventricular remodeling responses to IA/IG therapy. Furthermore, MRI is more sensitive in detecting subtle changes in the heart (53), but most studies use ultrasound. In future study designs, detailed quantitative assessment of the left ventricle using MRI should be included, as it offers superior spatial and contrast resolution, operator independence, and reproducibility. Moreover, based on NYHA functional classification and VO2 peak assessment, IA/IG treatment was also associated with sustained improvement in functional capacity and significant benefits in terms of improving patients’ quality of life.

In addition, the broader clinical implementation of IA has remained limited, partly because some clinicians still regard this treatment as too expensive. However, this view may overlook the substantially higher costs associated with heart transplantation (HTx), especially when long-term mechanical circulatory support is required as a bridge to HTx. From a health-economic perspective, IA/IG may represent a potentially cost-effective organ-preserving strategy for selected patients with DCM, particularly those with functionally pathogenic autoantibodies (44). Moreover, IA has already been incorporated into the guidelines of the American Society for Apheresis (ASFA) for idiopathic DCM, where it received a Grade B recommendation with the remark that it “can be applied without reservation in most patients in most circumstances.” (54) Nevertheless, the adoption of IA in routine clinical practice remains limited, underscoring the need for greater awareness of its potential role in appropriately selected patients. Looking ahead, novel antibody-targeting approaches may further broaden the therapeutic options for DCM. For example, small soluble molecules such as aptamers have been proposed as potential tools to specifically neutralize pathogenic autoantibodies, including *β*1AR-AABs. In theory, this strategy could offer a simpler, less time-consuming, and potentially less expensive alternative to IA (55). However, such approaches remain in an exploratory stage, and further preclinical and clinical studies are required to clarify their efficacy, safety, and optimal indications.

This study included different types of immunoadsorption columns for treatment, including protein A adsorption columns, tryptophan adsorption columns, and peptide-specific adsorption columns. These adsorption columns differ in terms of their mechanisms of action and therapeutic effects. Protein A adsorption columns, as the earliest type used in clinical applications, primarily function by binding to the Fc segment of IgG (56, 57). The reusable nature of protein A adsorption columns makes them cost-effective, and they are therefore widely used in clinical settings. The tryptophan adsorption column can specifically target IgG3 through hydrophobic interactions and has a higher clearance rate for IgG3 (58). However, its high cost and single-use nature may limit its clinical application. Danel et al. also used peptide-specific adsorption columns in their study (14, 22). However, there were no differences between specific IA and non-specific IA in terms of cardiac function and patient prognosis in DCM patients or adverse reactions. Despite the advantage of targeted removal of pathogenic antibodies, the development and clinical application of specific IA remain significantly limited due to the lack of large-scale clinical data. The differences in the mechanisms of action of different adsorption columns provide a theoretical basis for individualized treatment. Future research may explore the optimal strategy for combining adsorption columns, establish patient-specific IA treatment selection criteria based on antibody profiles, and extend follow-up periods to assess the long-term remodeling effects on cardiac structure and function.

This review has several limitations. First, only two of the included studies were RCTs, and non-RCT studies may reduce the reliability of the results. Additionally, all studies were conducted using an open-label design, as conducting a blinded study was difficult due to the invasive nature of the interventions. Furthermore, most studies did not provide detailed reporting on randomization and allocation concealment, which may reduce the robustness of the results. Second, most studies were conducted in Germany, and the study population's homogeneity may limit the conclusions’ generalizability. Additionally, differences in the type of immunadsorption columns, IG dosage, and treatment duration may also influence the results. Therefore, the generalizability of our findings requires further validation. Finally, the lack of published clinical trials on IA/IG treatment for DCM limited the number of studies we could include. Currently, a multicenter, randomized, double-blind, prospective study on IA/IG treatment for DCM is in progress (IASO-DCM), with patients undergoing 24 months of long-term follow-up (59). We anticipate that this study will provide more high-quality validation of the long-term benefits of IA/IG treatment for DCM patients and standardized treatment protocols.

## Conclusion

In summary, IA/IG therapy for DCM patients is safe and can improve cardiac structure and function to a certain extent. These findings support IA/IG therapy as a promising method for slowing disease progression and improving quality of life in DCM patients. However, published clinical trials on IA/IG therapy for DCM remain limited. Current evidence supports IA/IG as a potential adjunctive therapy for DCM, capable of short-term improvements in cardiac function and clinical symptoms. However, its long-term benefits and standardized treatment protocols require further validation through high-quality studies. Future research directions should include optimizing treatment strategies, expanding population representativeness, and exploring the optimal role of IA/IG in the comprehensive management of DCM.

## Data Availability

The original contributions presented in the study are included in the article/Supplementary Material, further inquiries can be directed to the corresponding author.
