# Supplementary material for: Immunoadsorption and subsequent immunoglobulin G replacement (IA/IG) in patients with dilated cardiomyopathy: a systematic review and meta-analysis
Source: Front Cardiovasc Med. 2026 Jun 23;13:1840280. doi: 10.3389/fcvm.2026.1840280 (PMC13337766; doi:10.3389/fcvm.2026.1840280)
Supplement: Supplementary file 2 [file Datasheet1.pdf]

Search strategy

#1 dilated cardiomyopathy [MeSH Terms]

#2 dilated cardiomyopathy [Title/Abstract]

#3 cardiomyopathy [MeSH Terms]

#4 cardiomyopathy [Title/Abstract]

#5 #1 OR #2 OR #3 OR #4

#6 Immunosorption [MeSH Terms]

#7 Immunosorption [Title/Abstract]

#8 Immunoglobulin [MeSH Terms]

#9 Immunoglobulin [Title/Abstract]

#10 IA/IG [MeSH Terms]

#11 IA/IG [Title/Abstract]

#12 Immunotherapy [MeSH Terms]

#13 Immunotherapy [Title/Abstract]

#14 #6 OR #7 OR #8 OR #9 OR #10 OR #11 OR #12 OR #13

#15 trial [MeSH Terms]

#16 trial [Title/Abstract]

#17 #15 OR #16 OR

#18 #5 AND #14 AND #17
