# Supplementary figures and images for: Immunoadsorption and subsequent immunoglobulin G replacement (IA/IG) in patients with dilated cardiomyopathy: a systematic review and meta-analysis
Source: Front Cardiovasc Med. 2026 Jun 23;13:1840280. doi: 10.3389/fcvm.2026.1840280 (PMC13337766; doi:10.3389/fcvm.2026.1840280)

## LVEF (A)

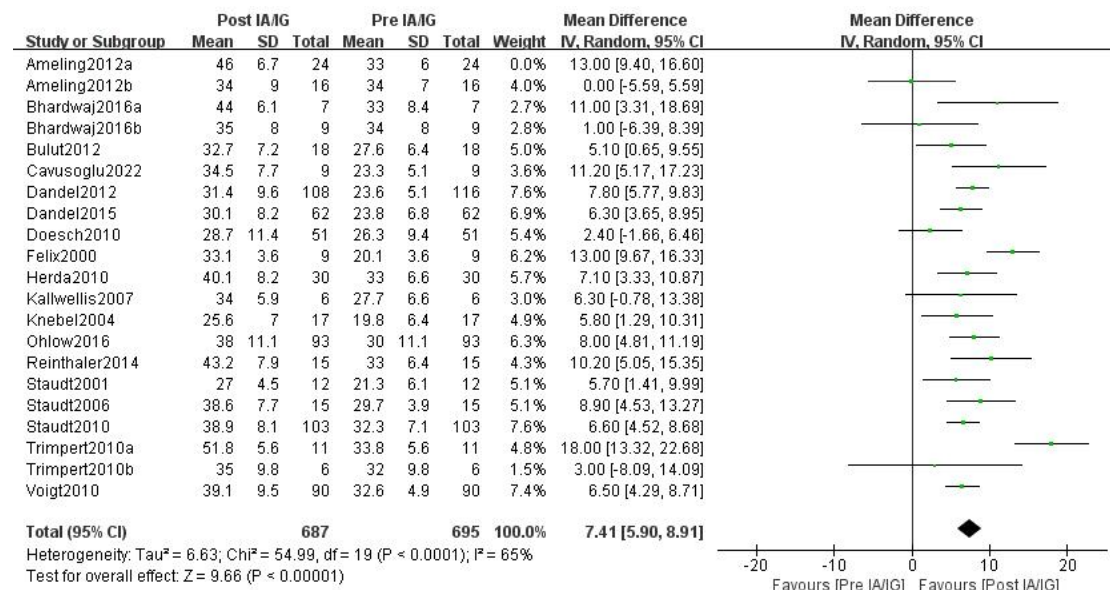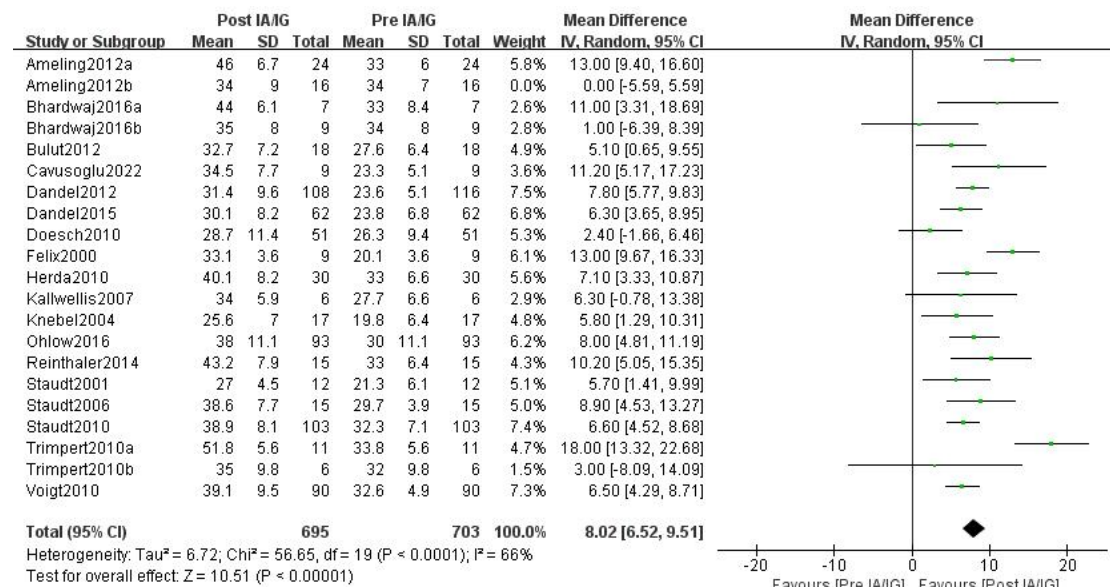

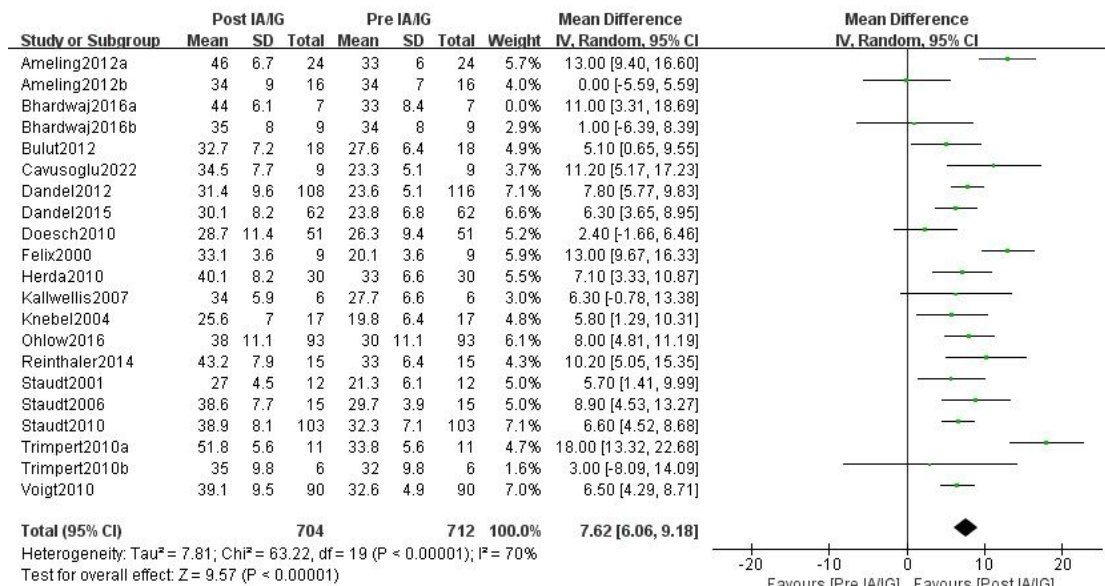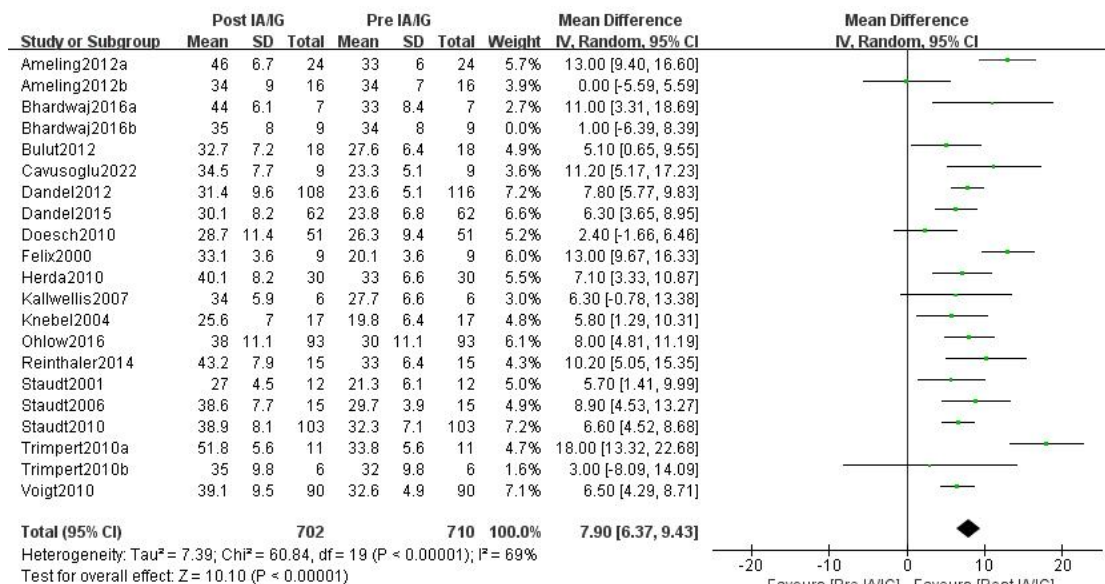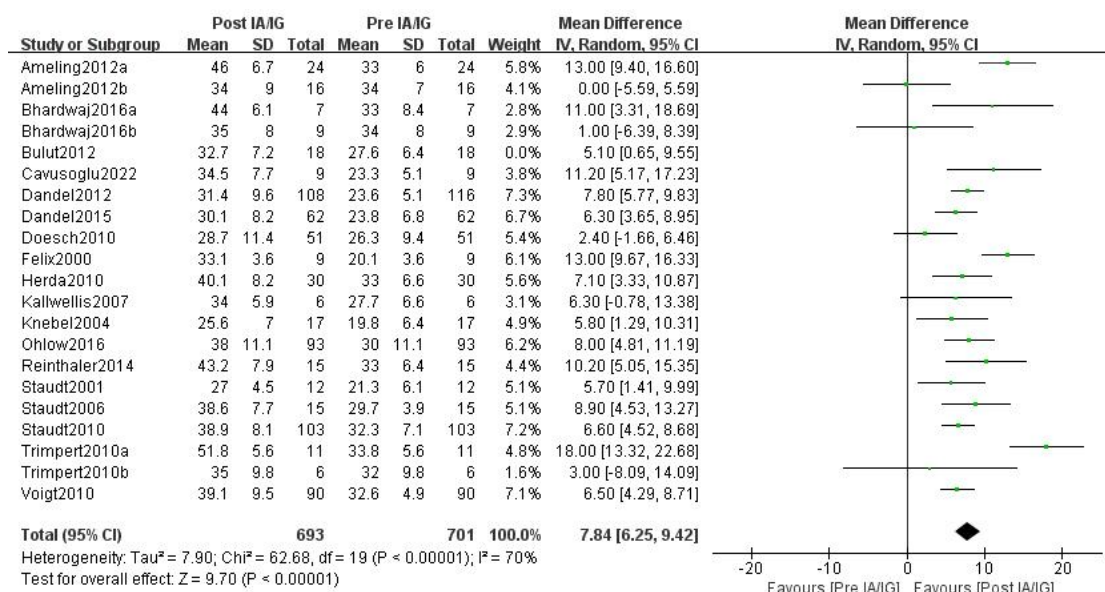

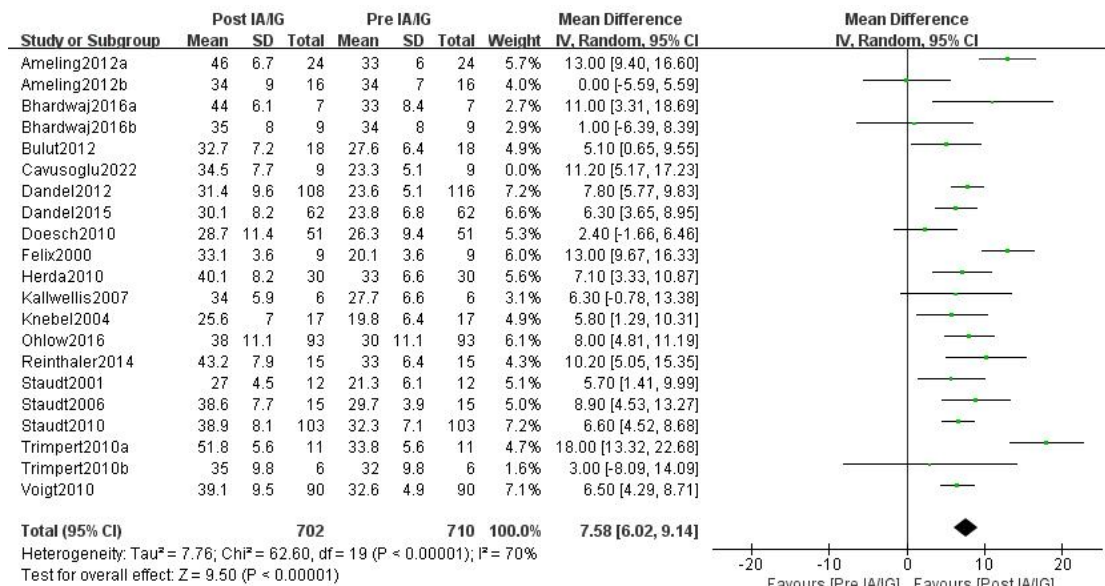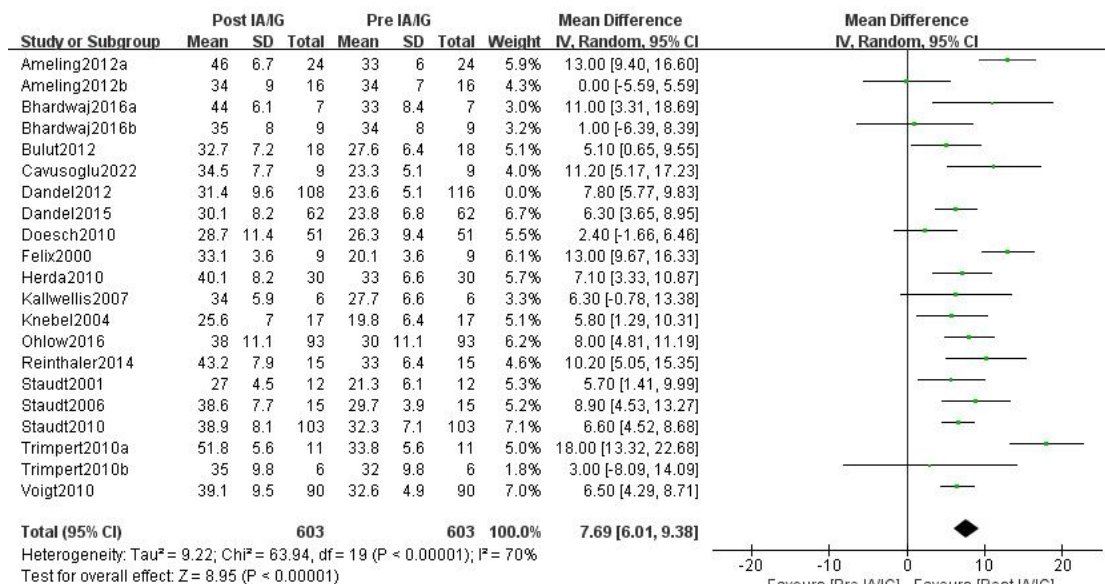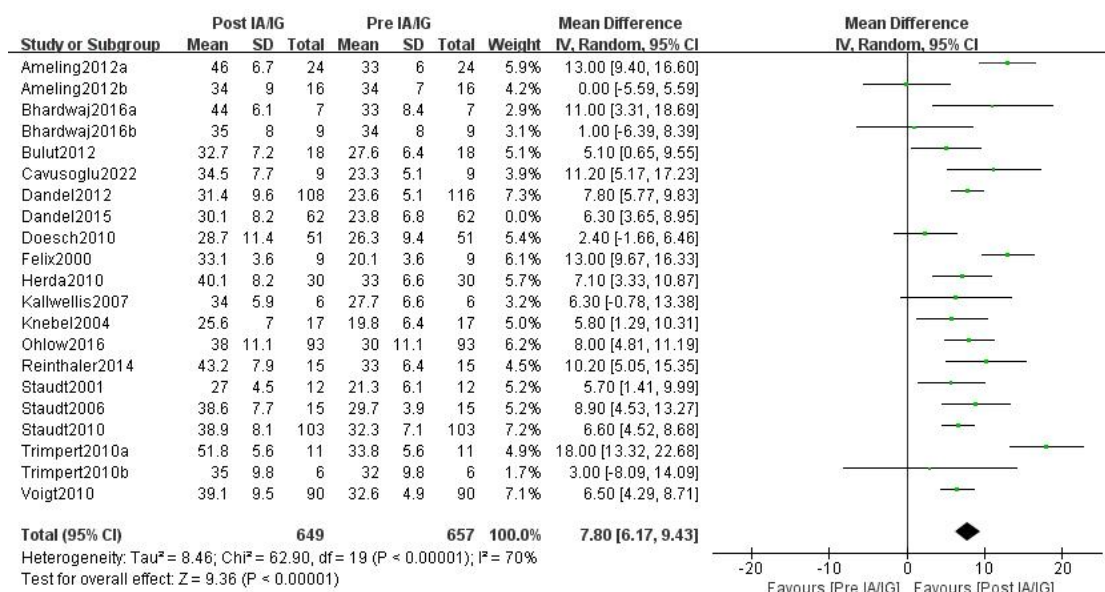

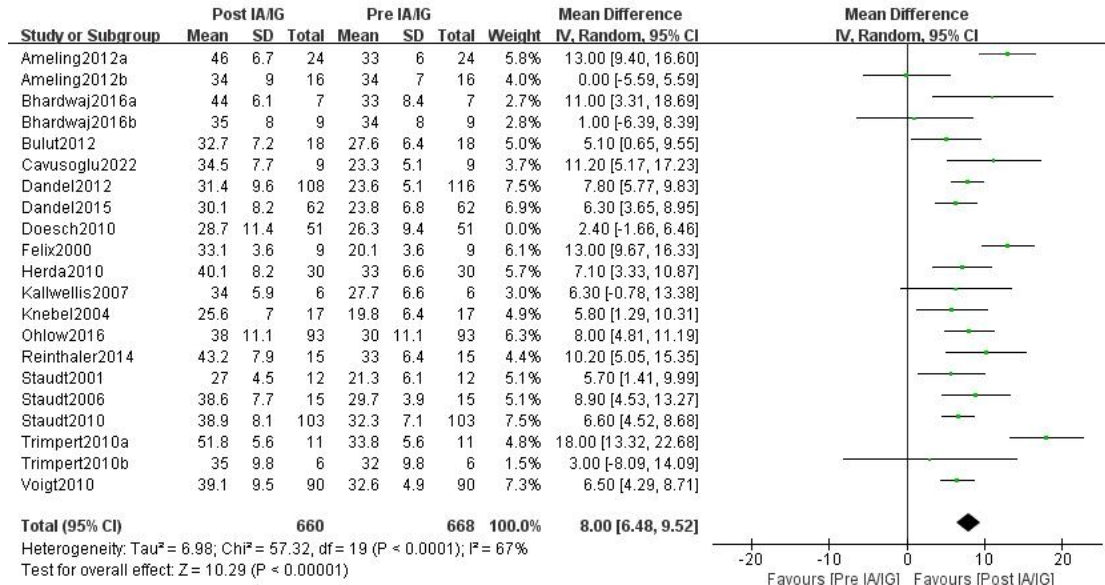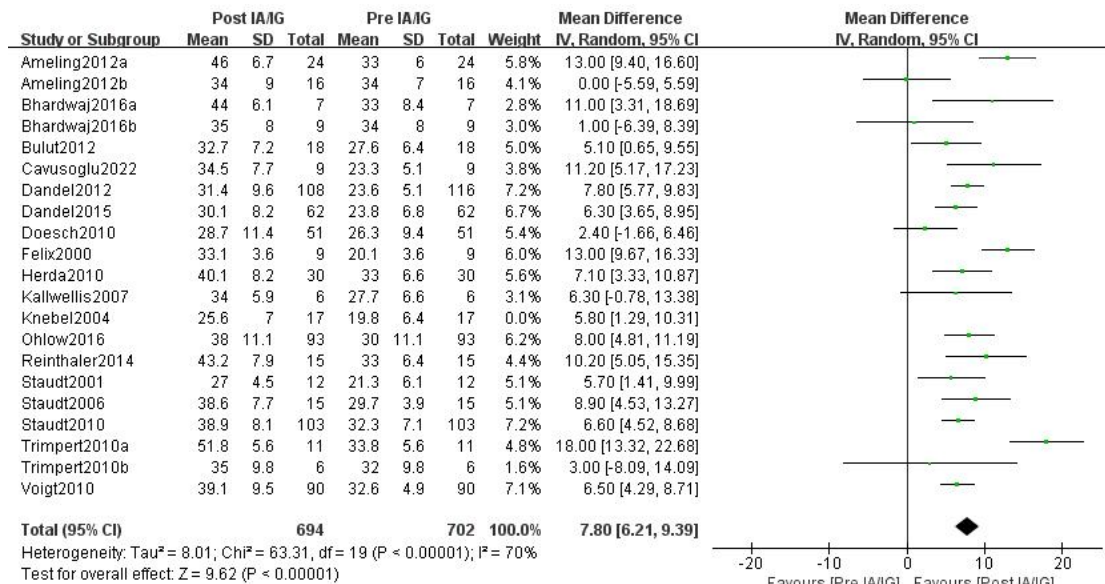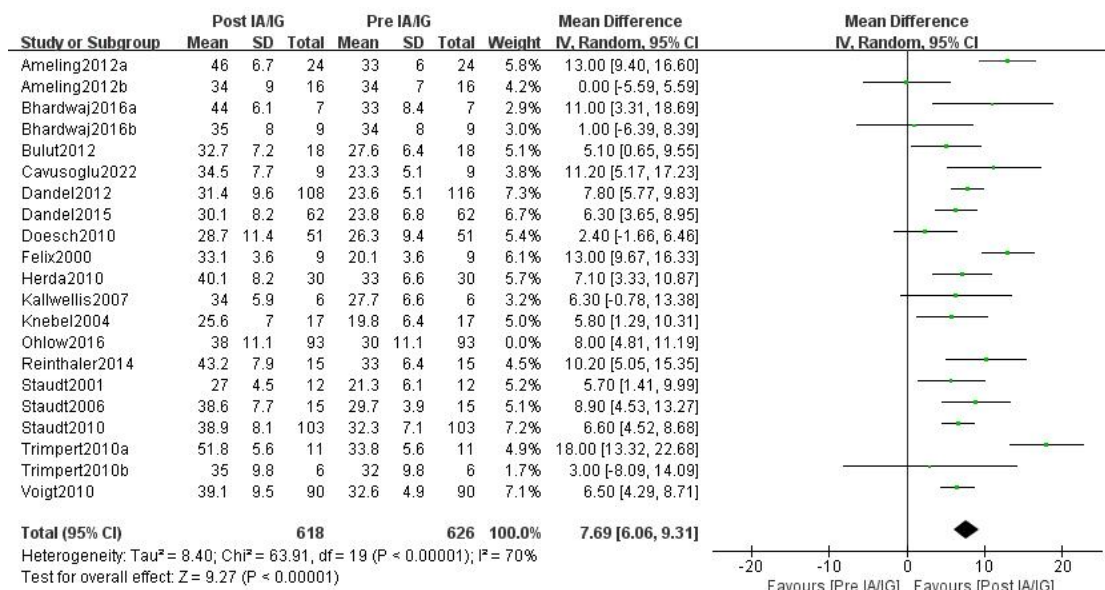

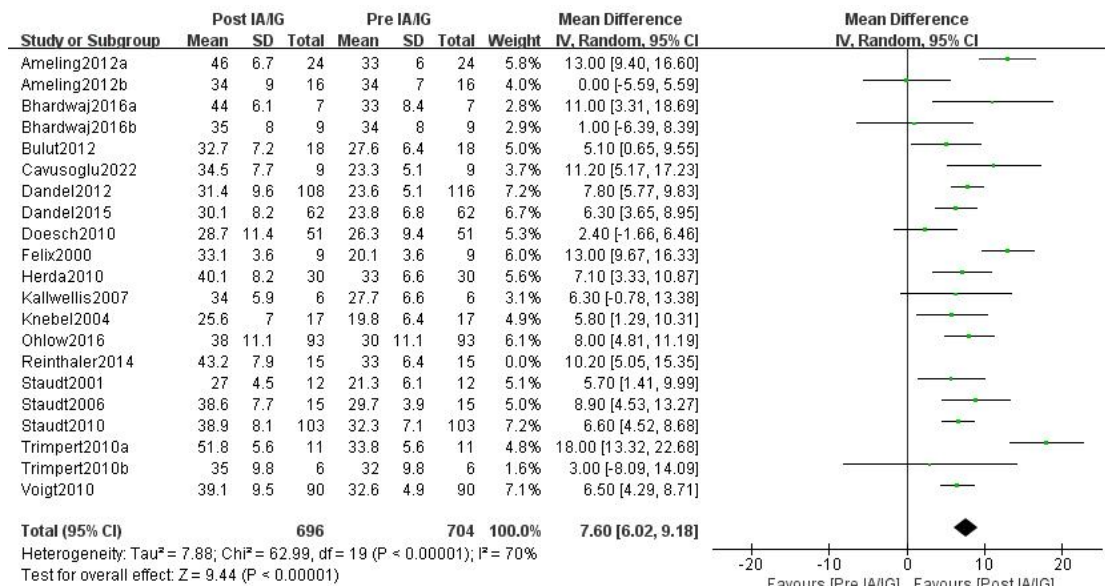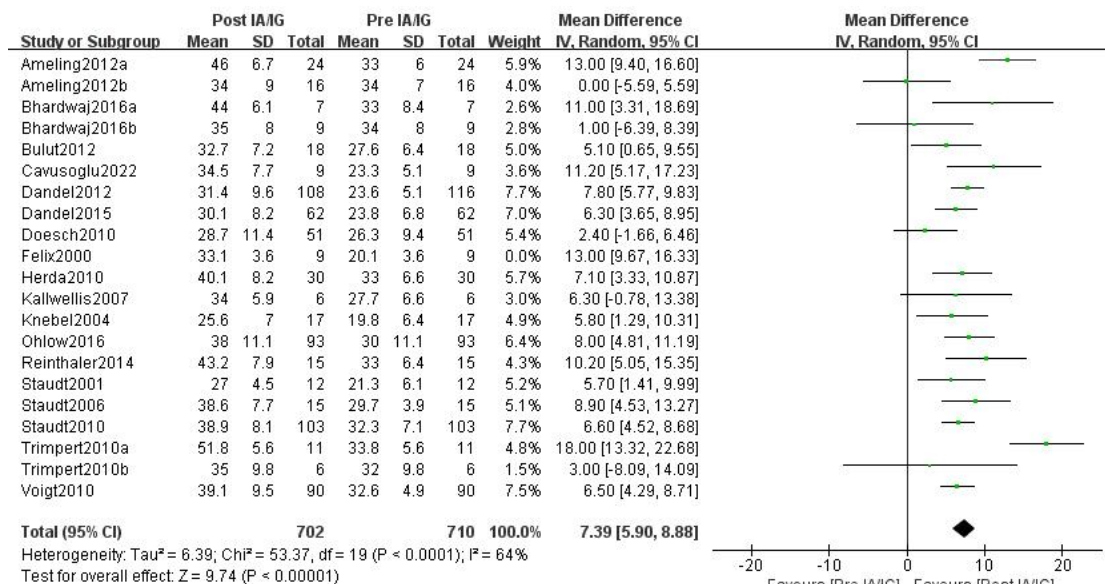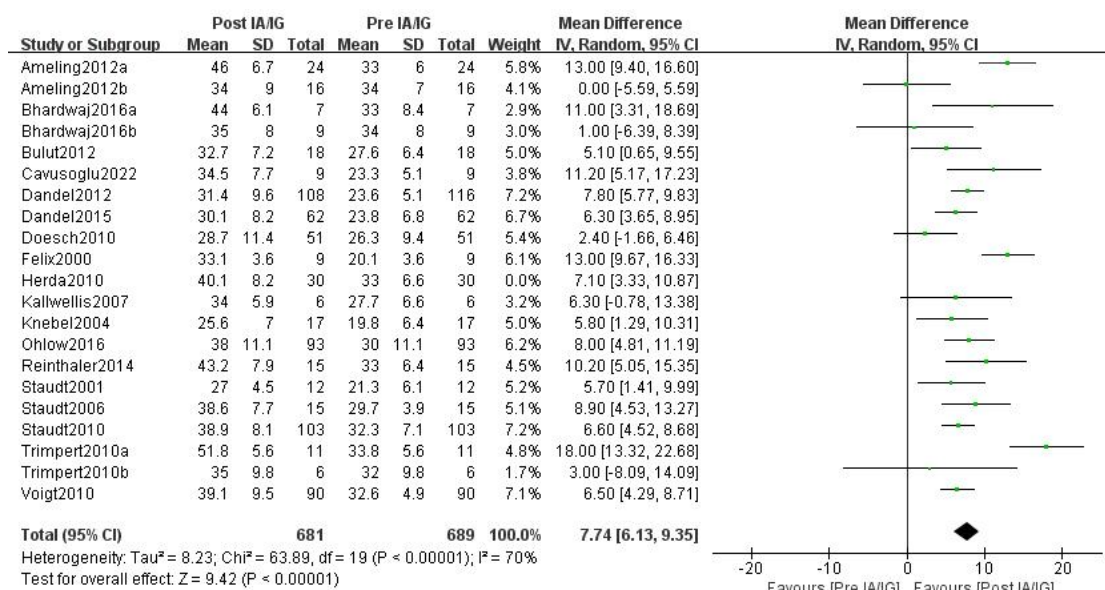

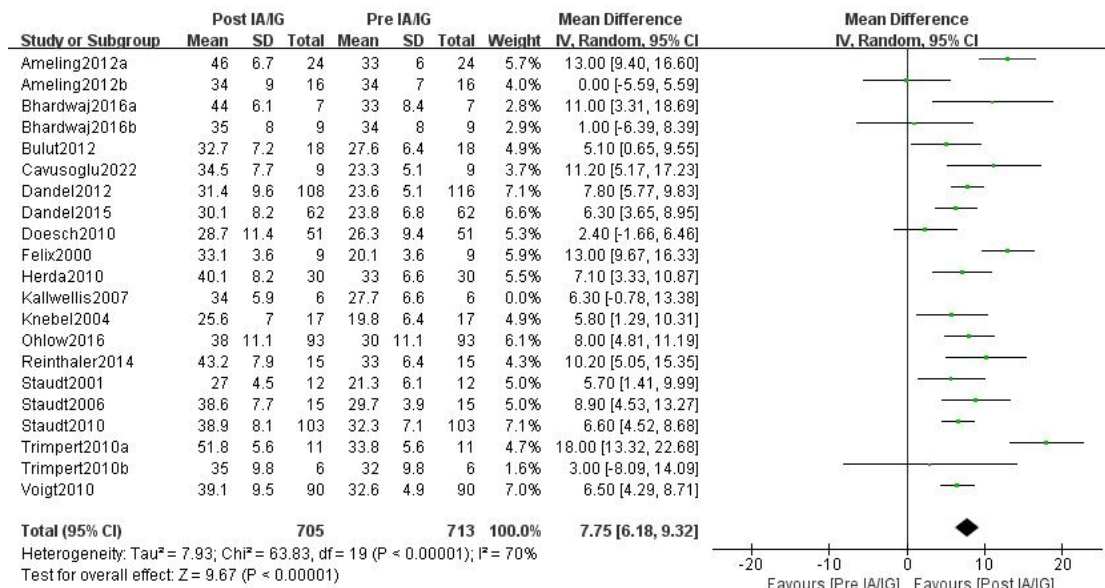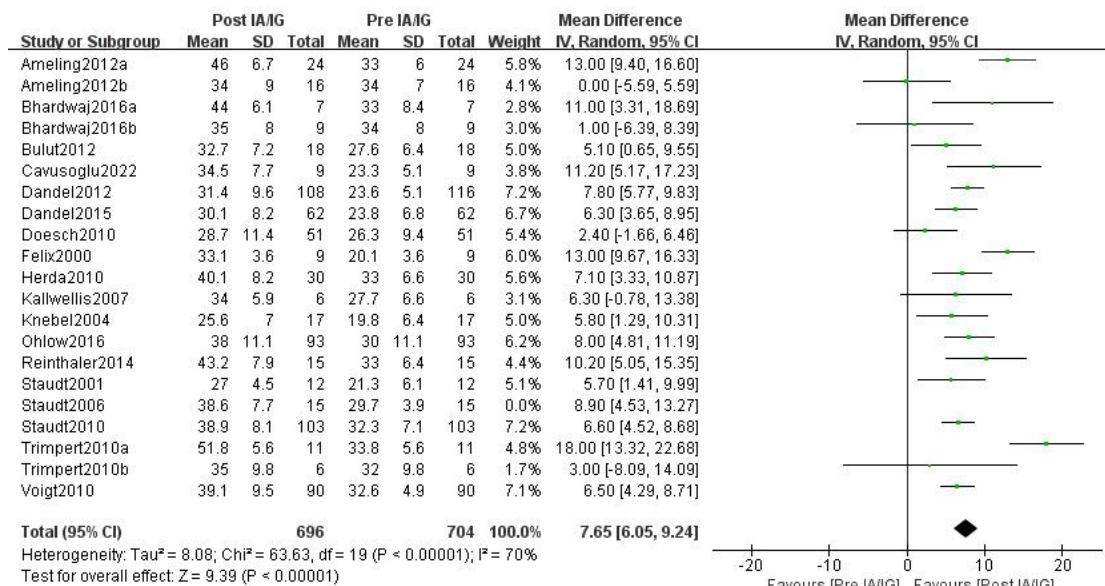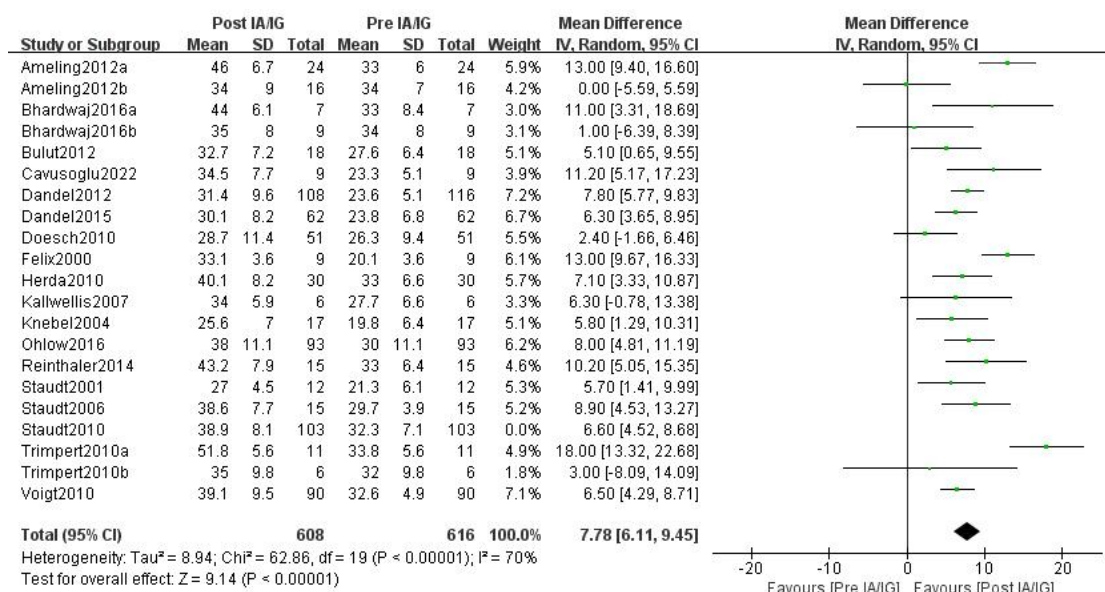

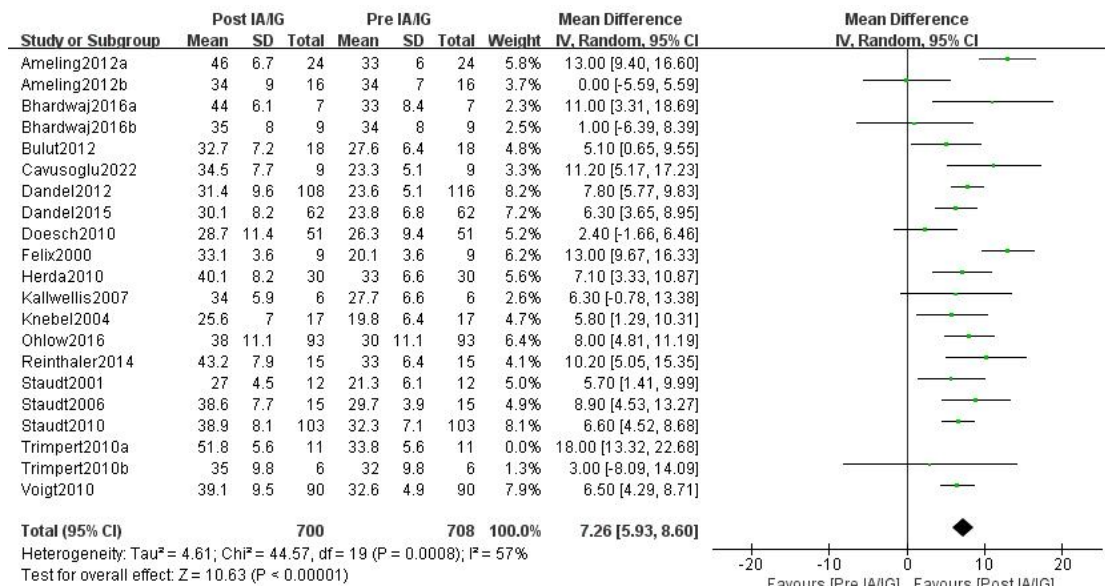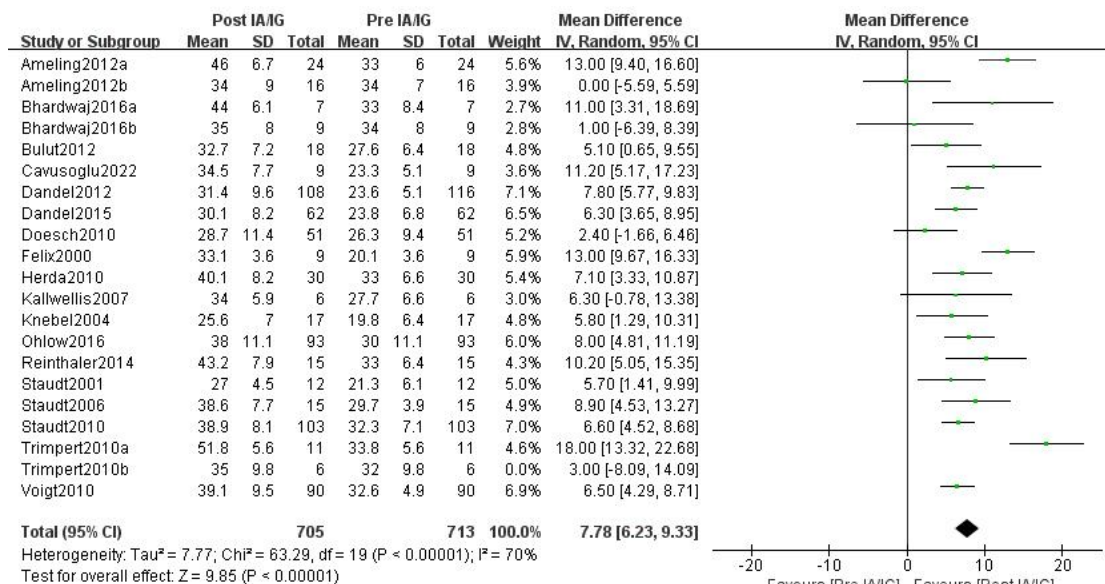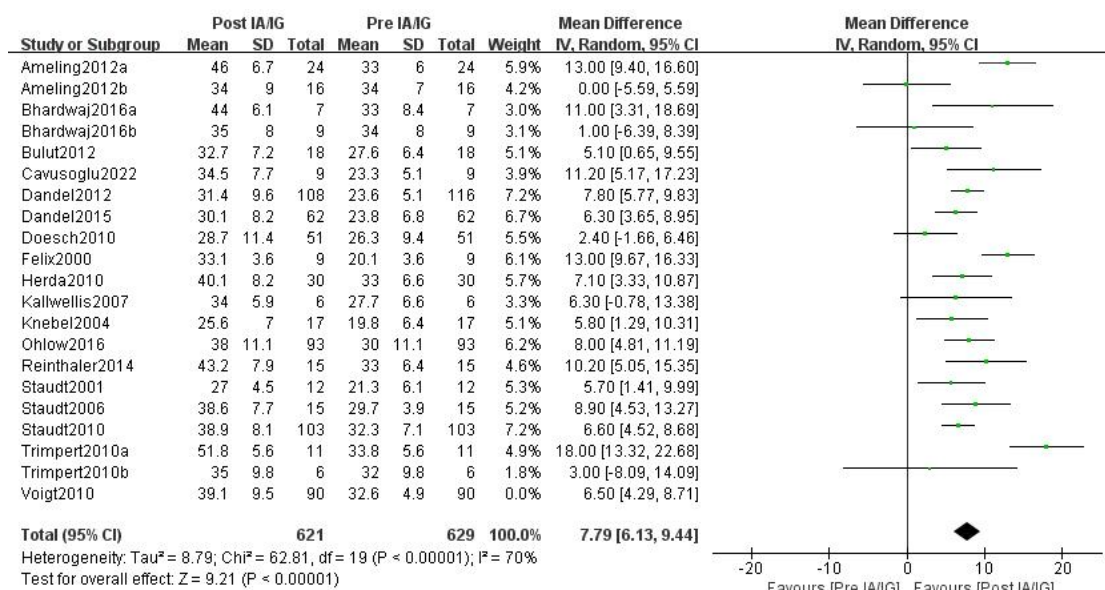

## LVEF (B)

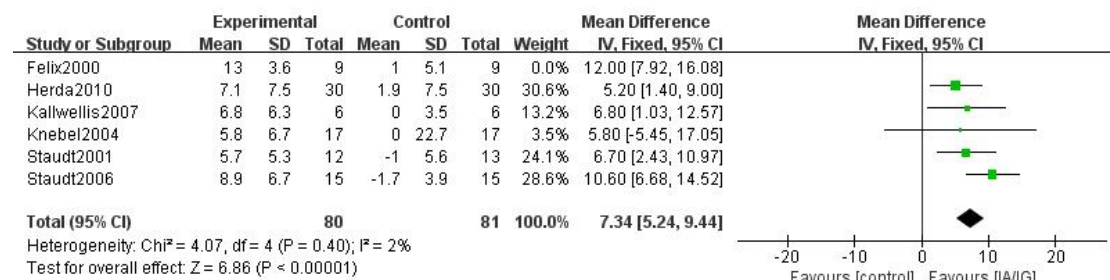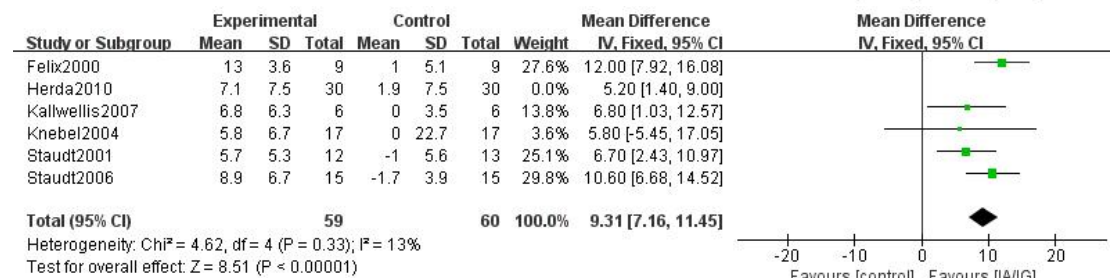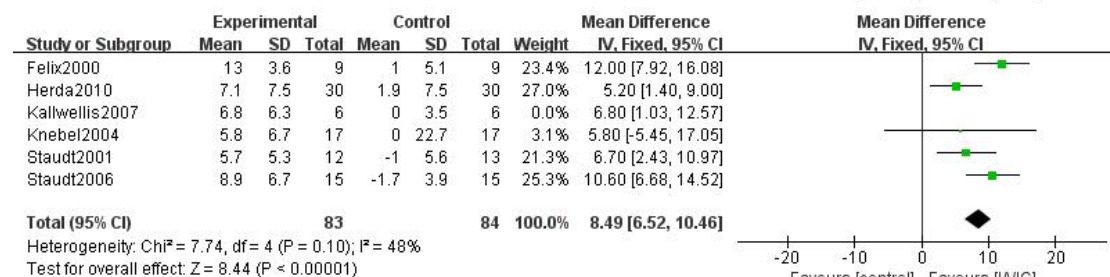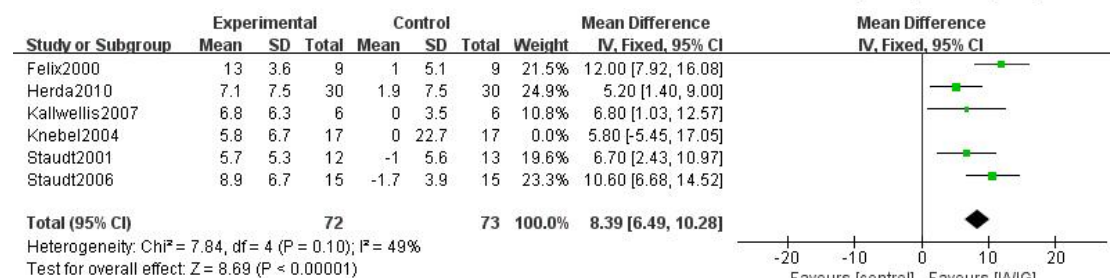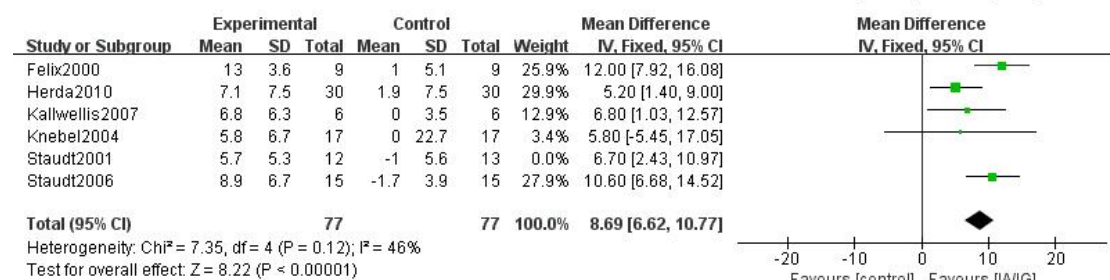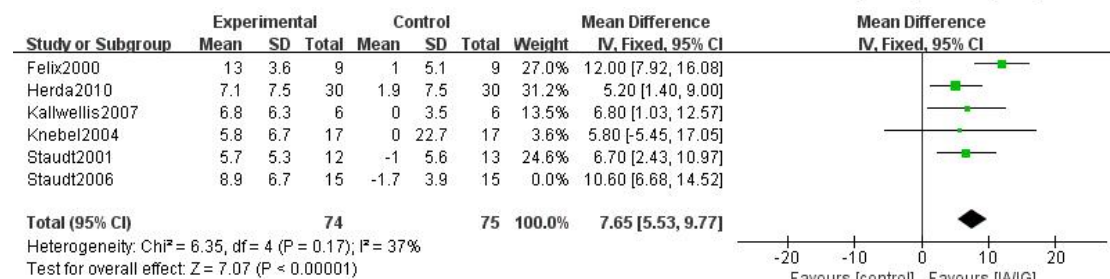

## LVEDD (A)

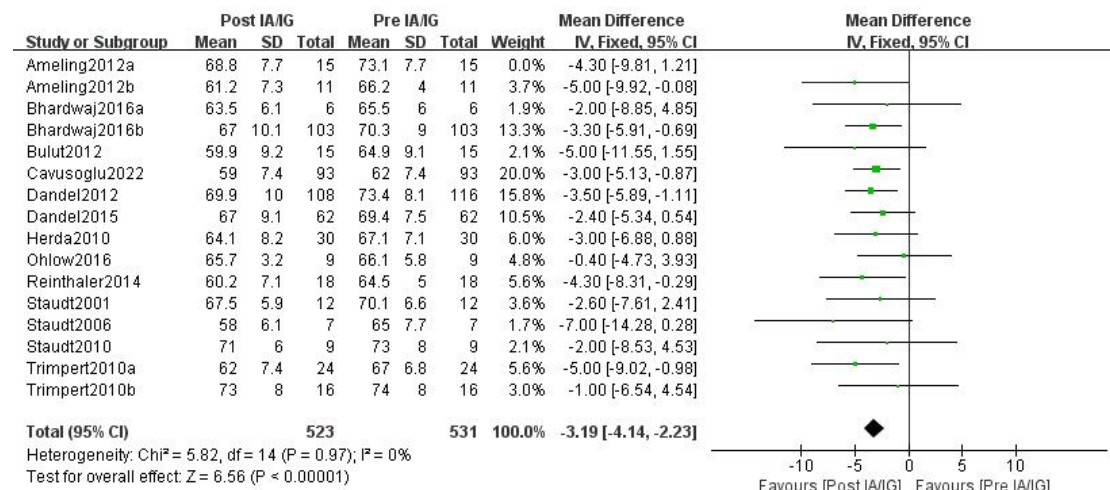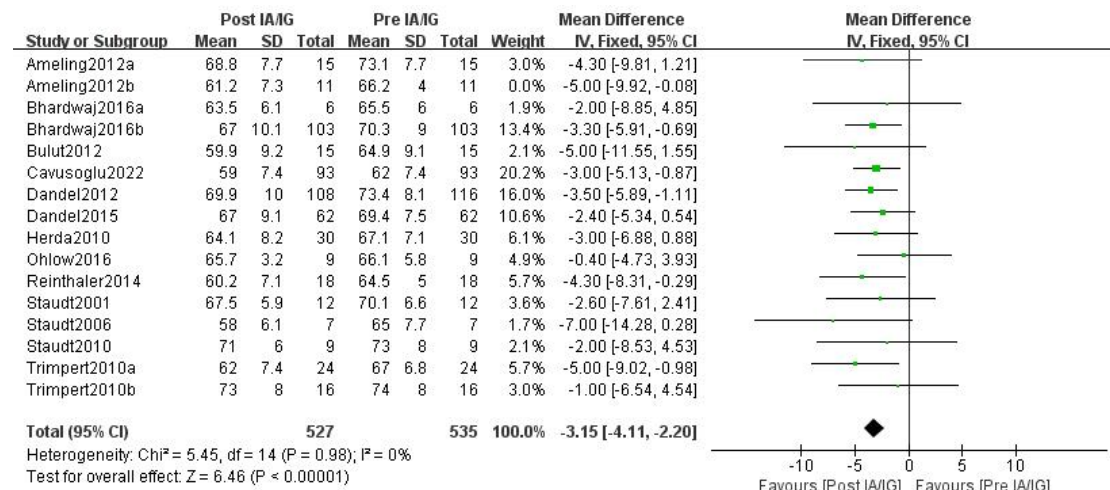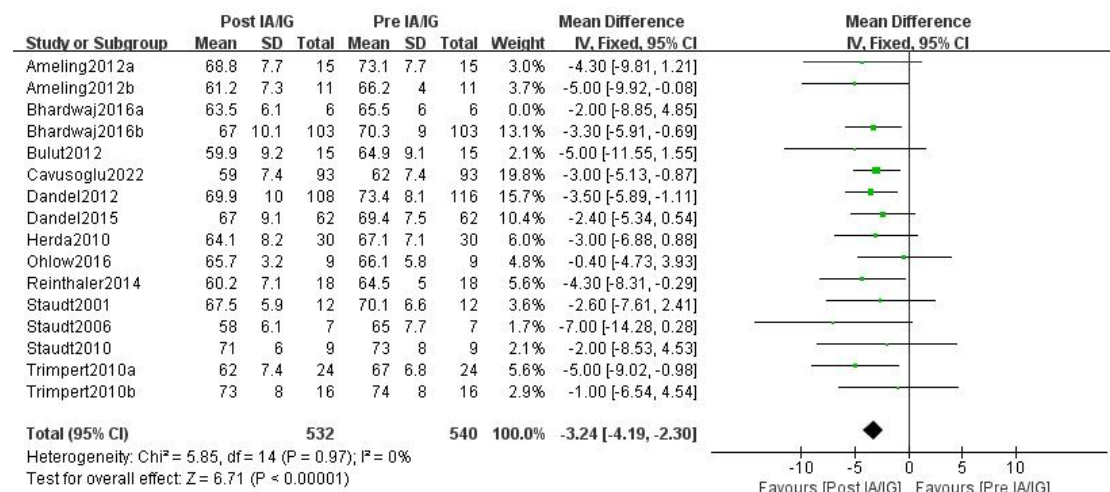

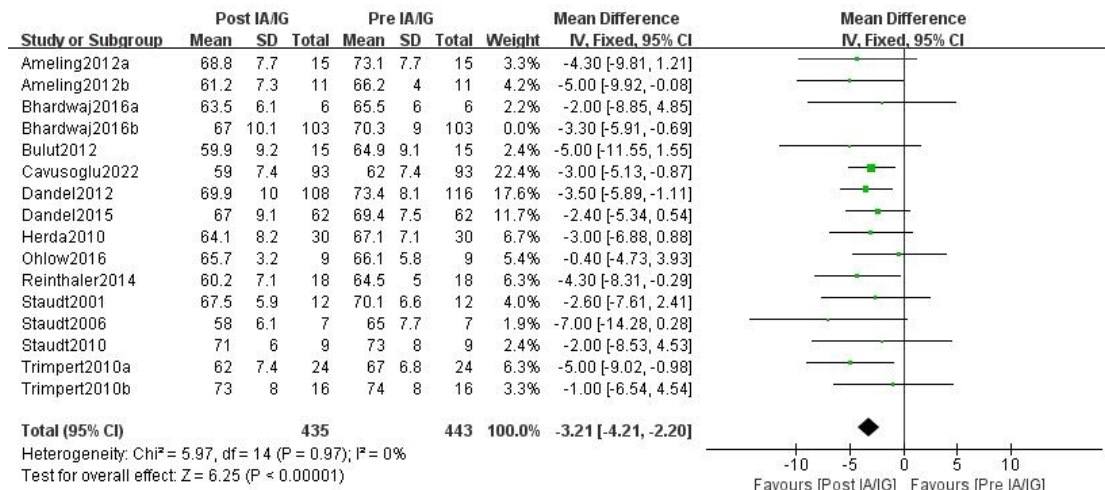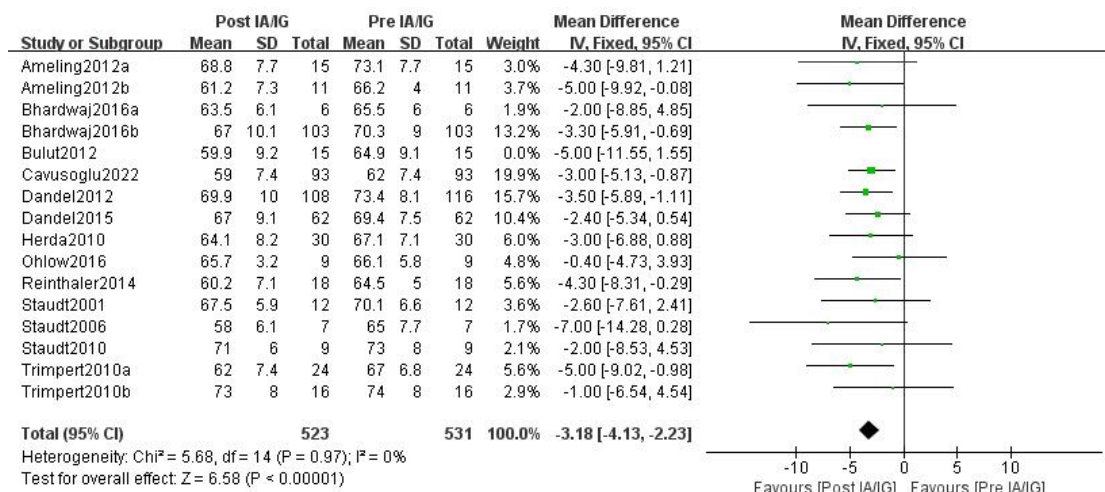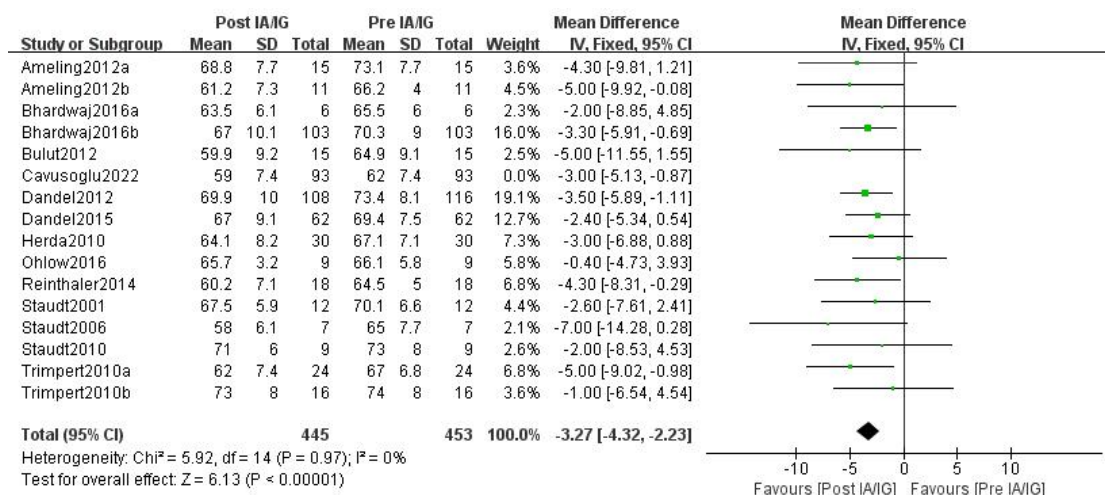

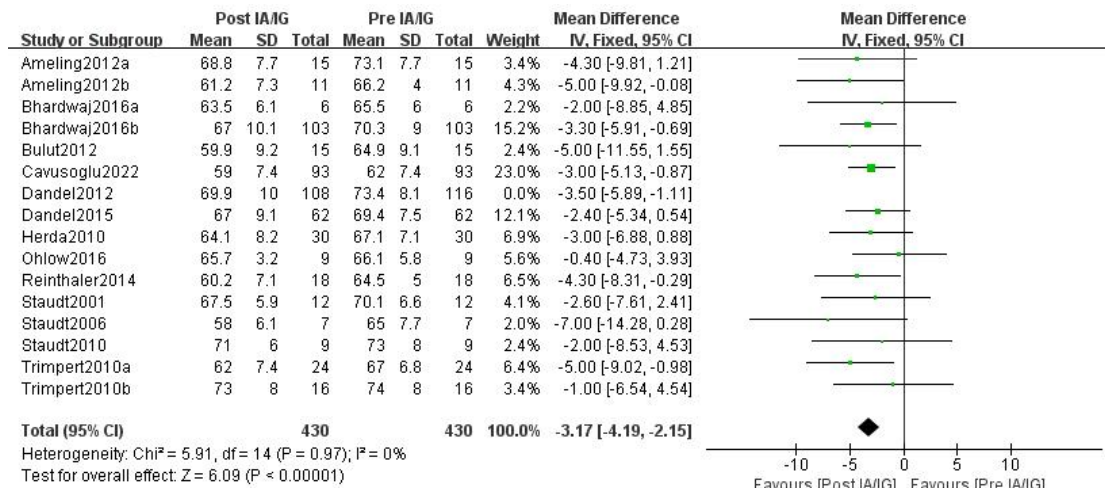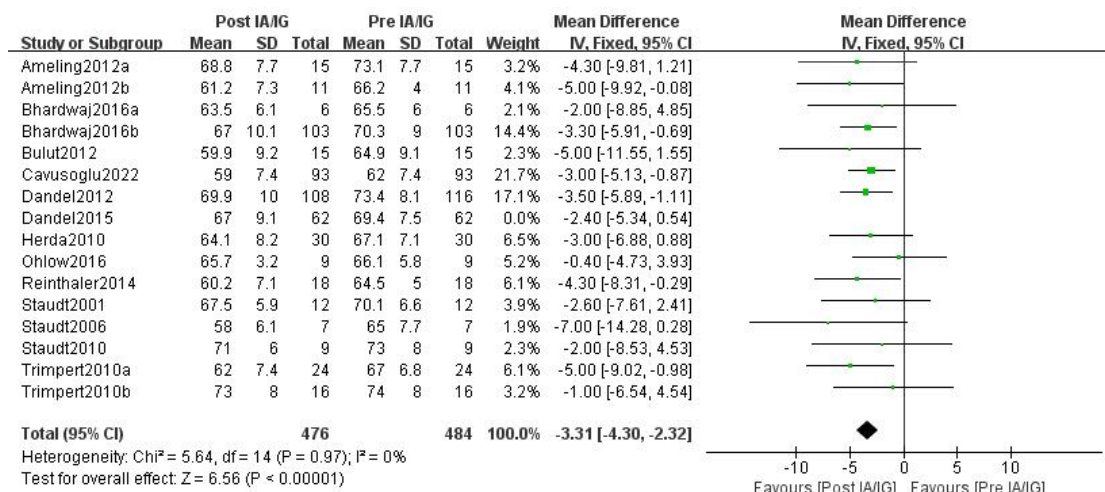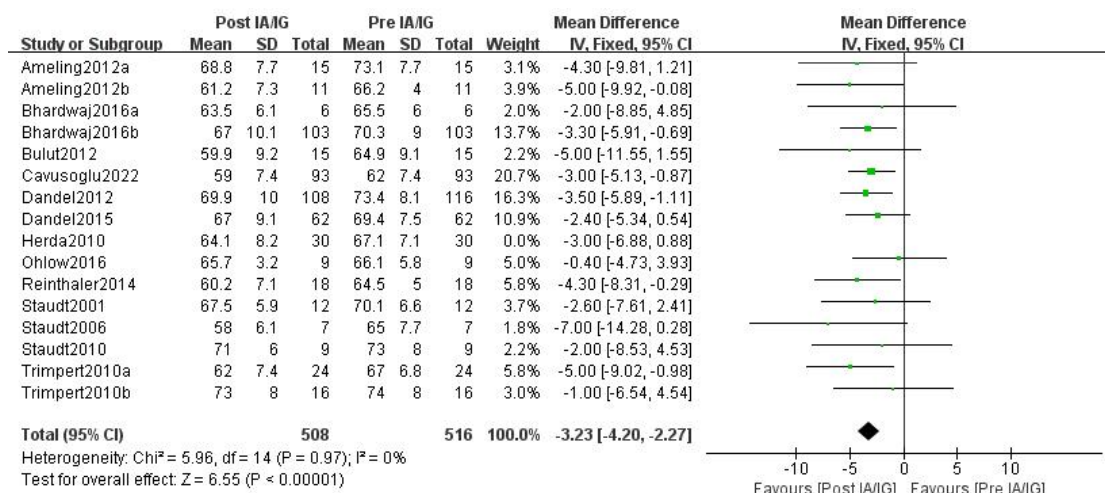

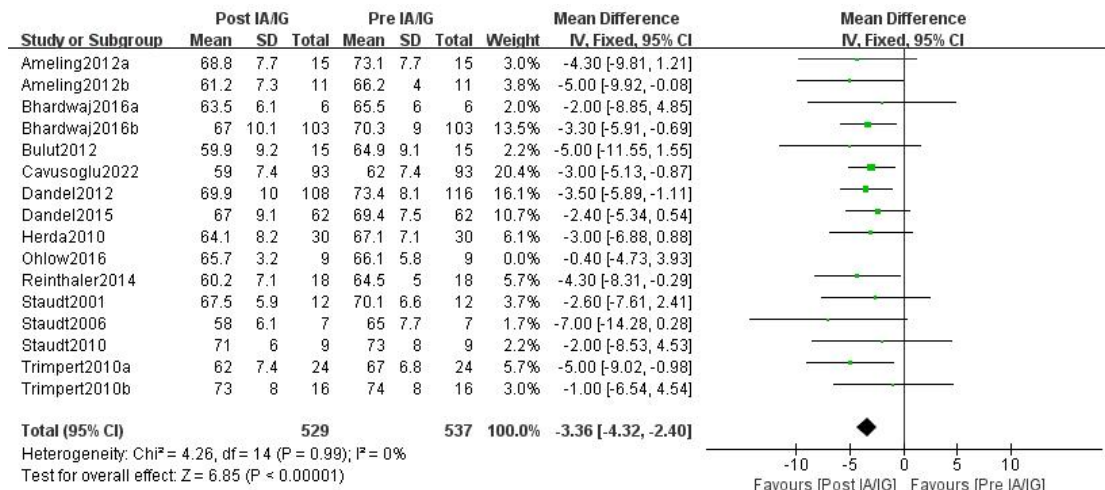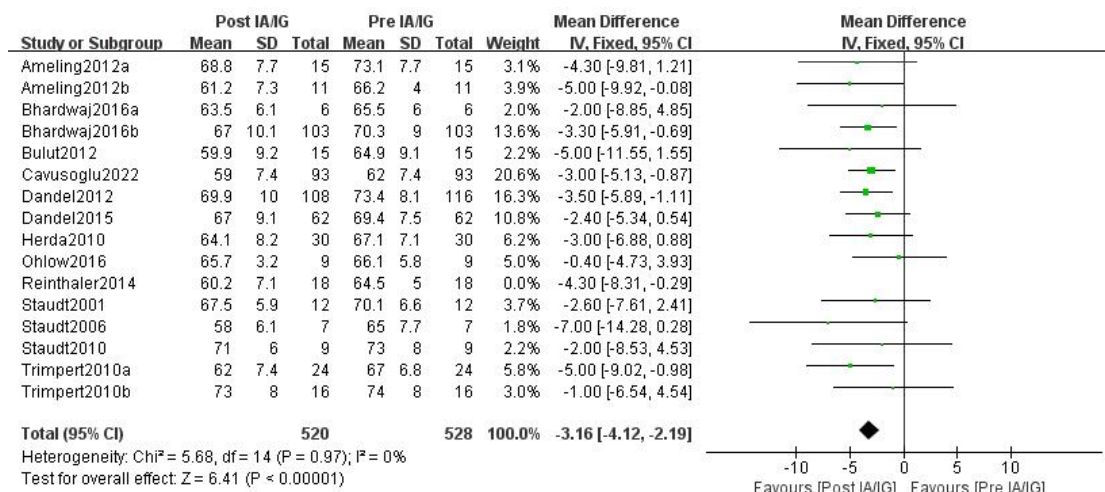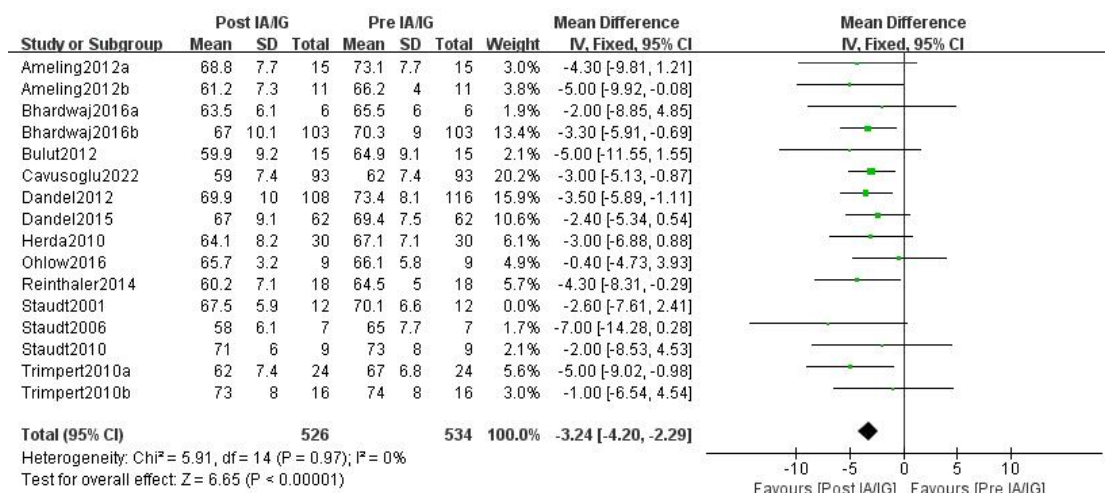

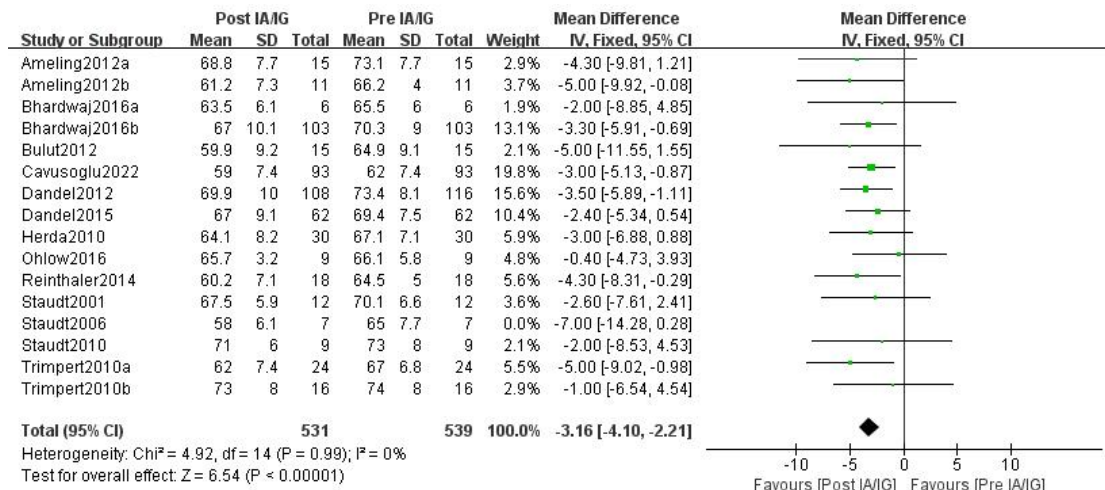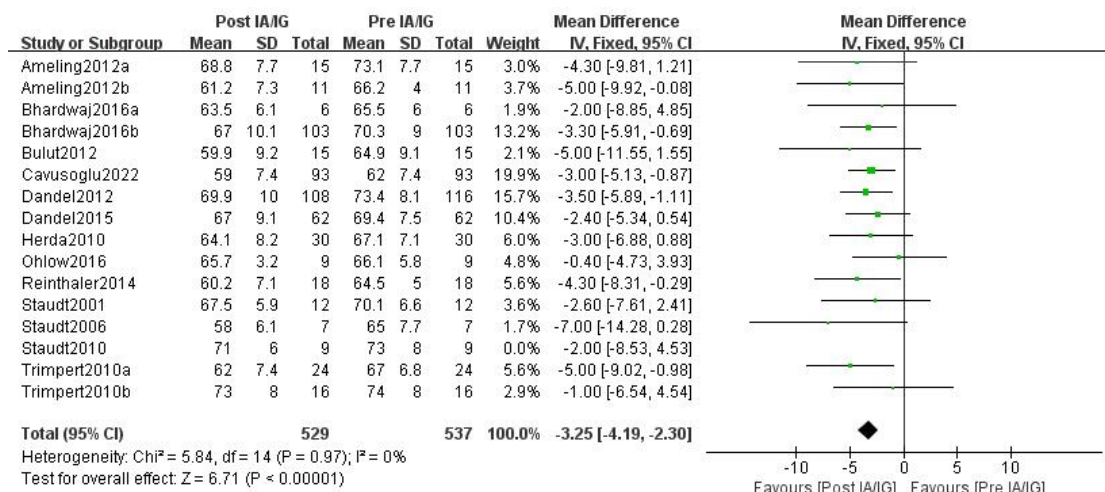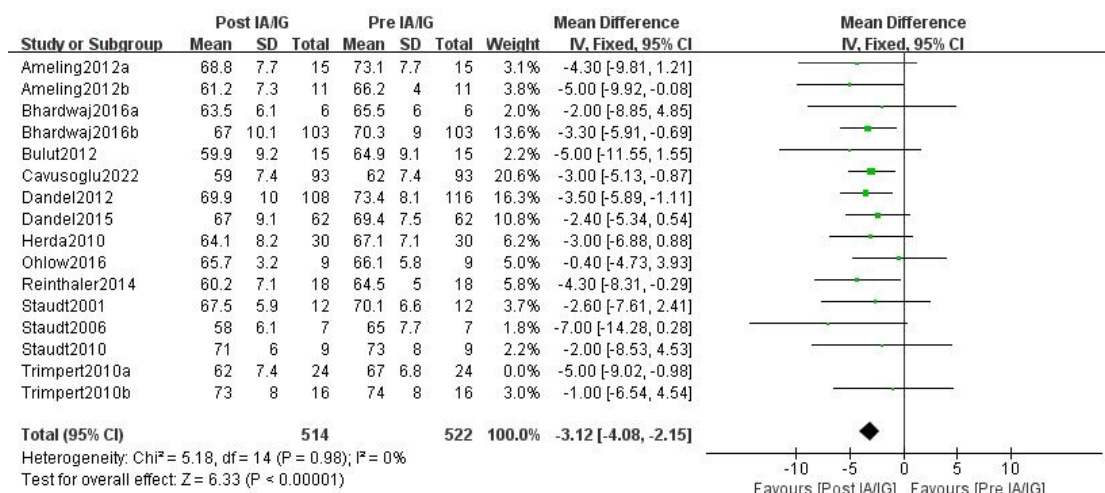

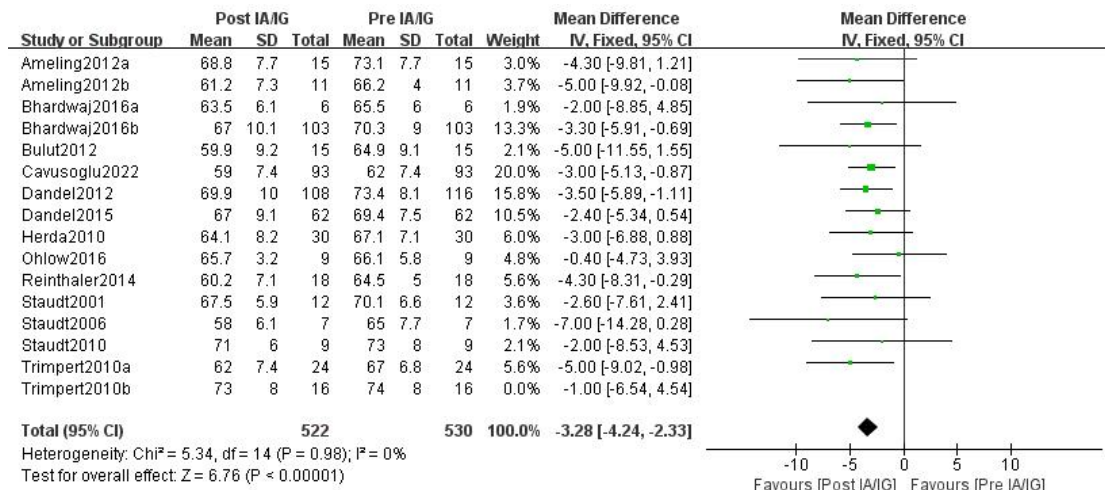

## LVEDD (B)

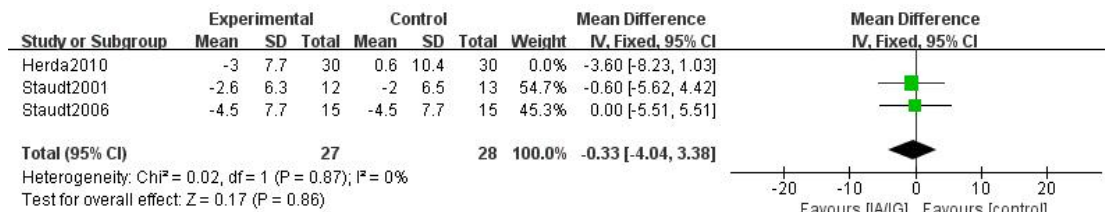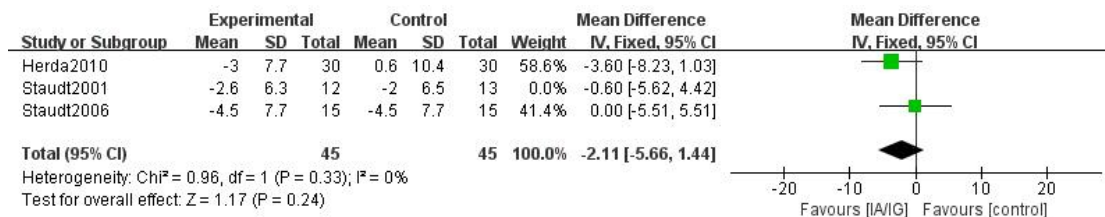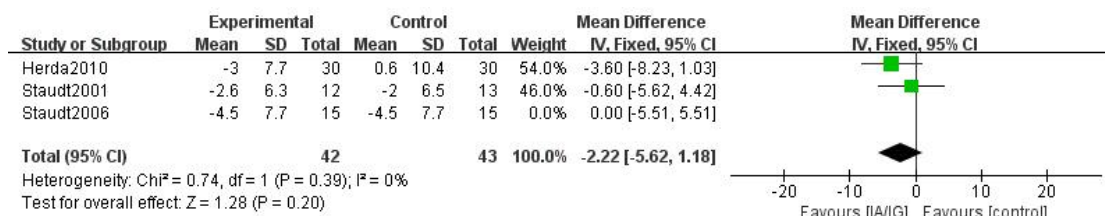

## NYHA classification

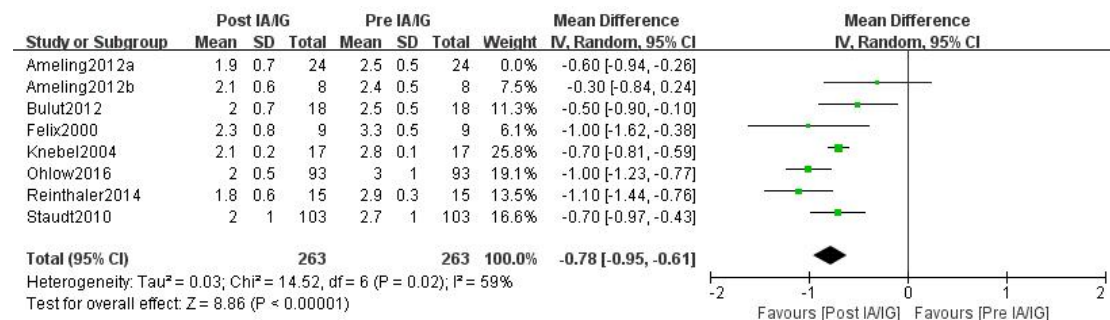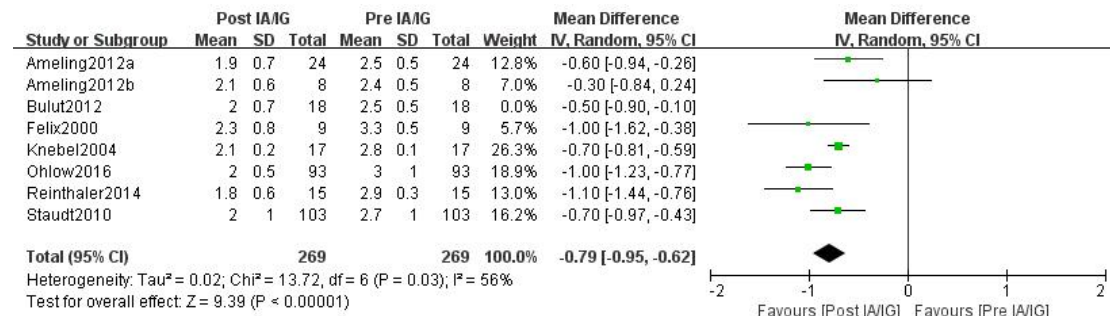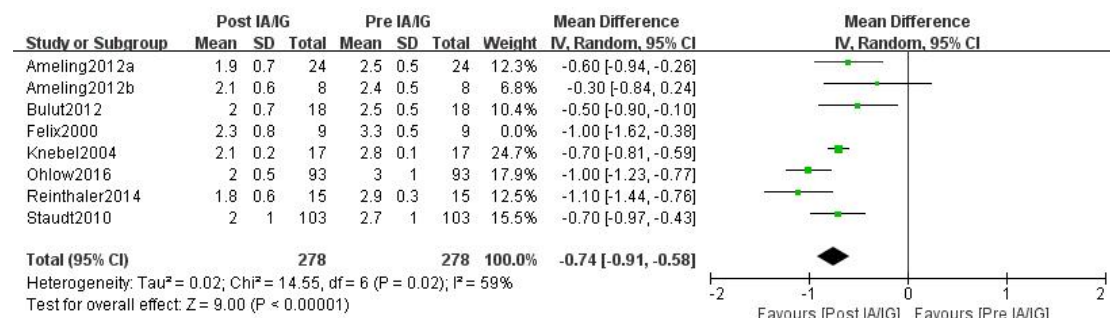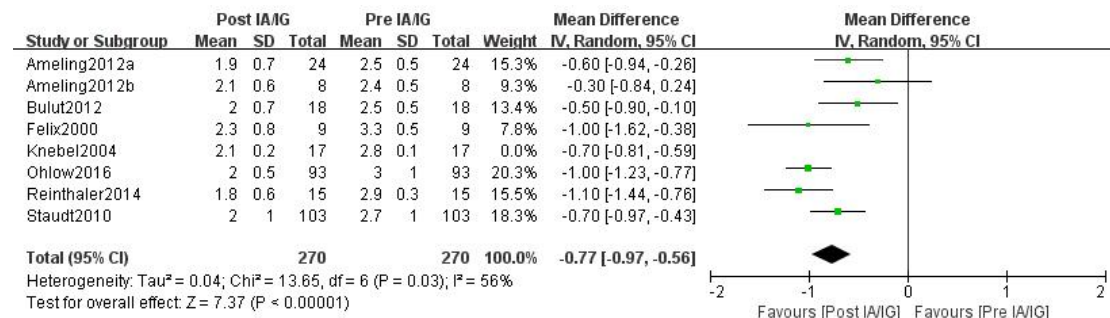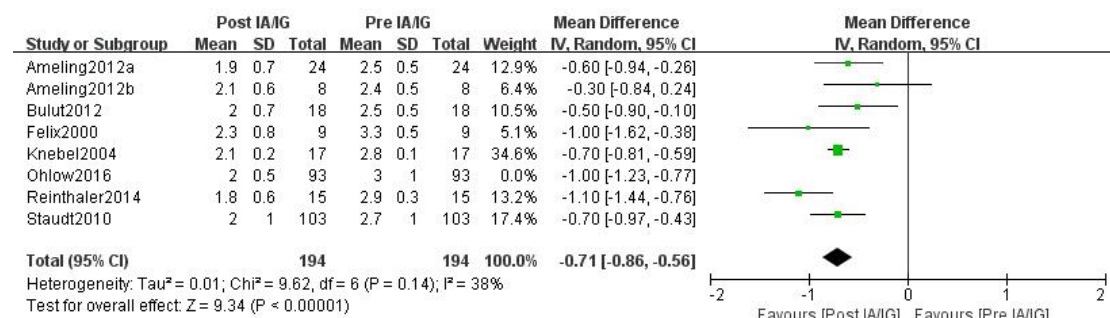

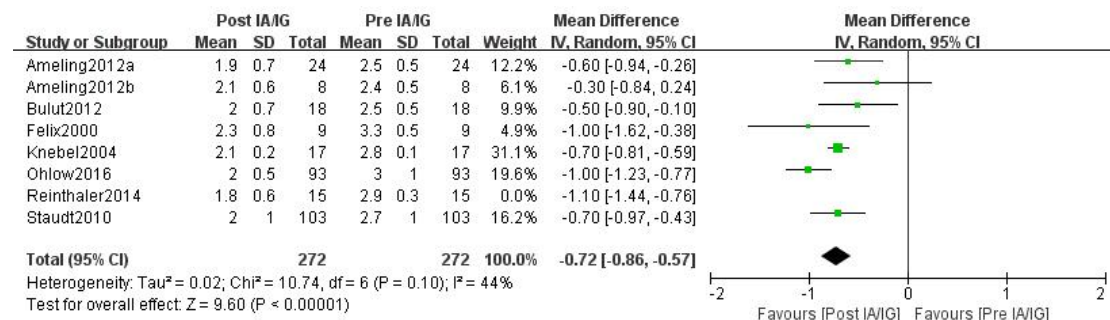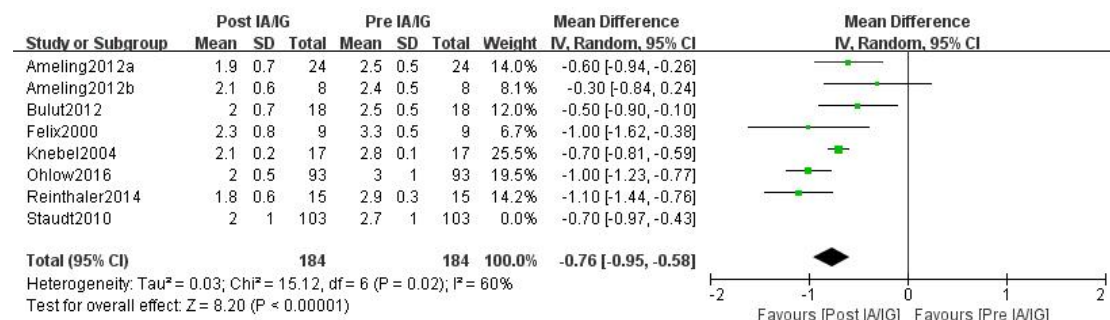

## VO2 peak

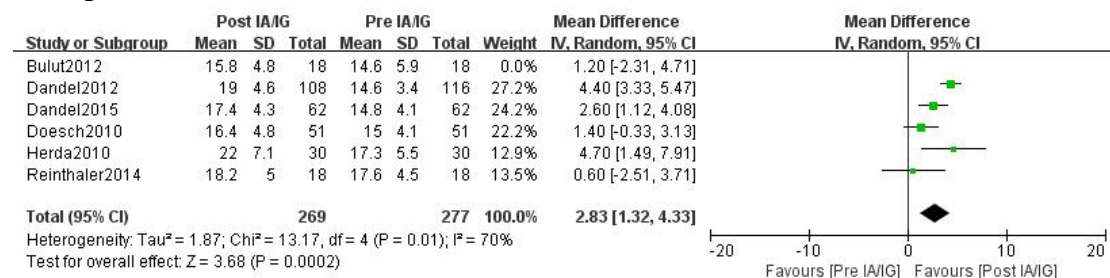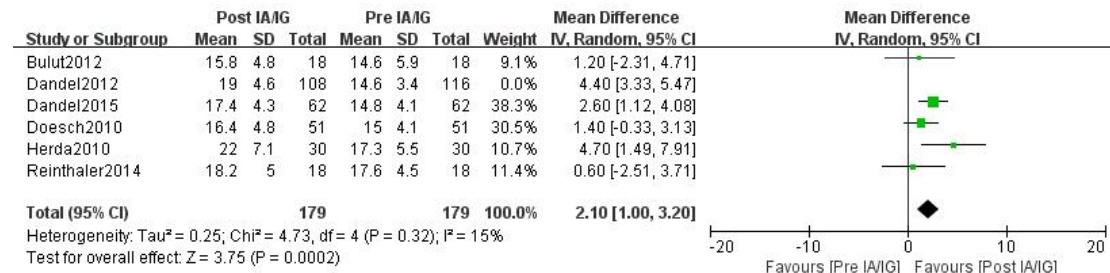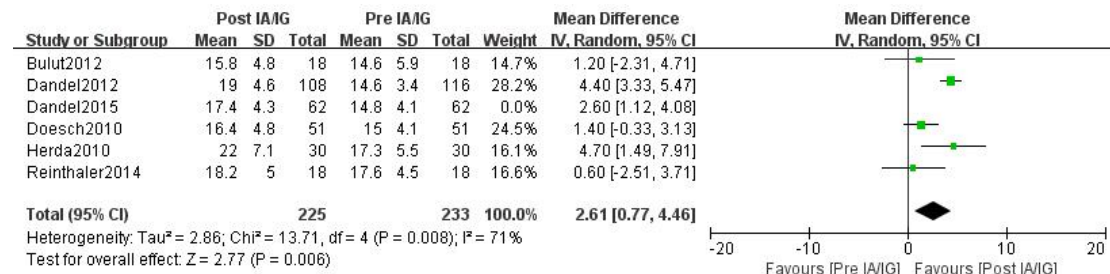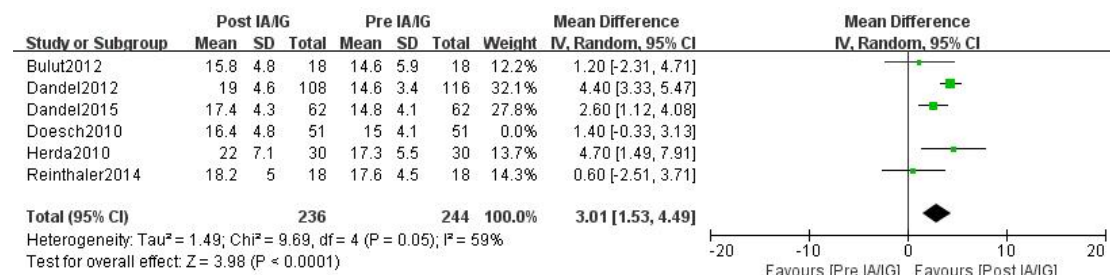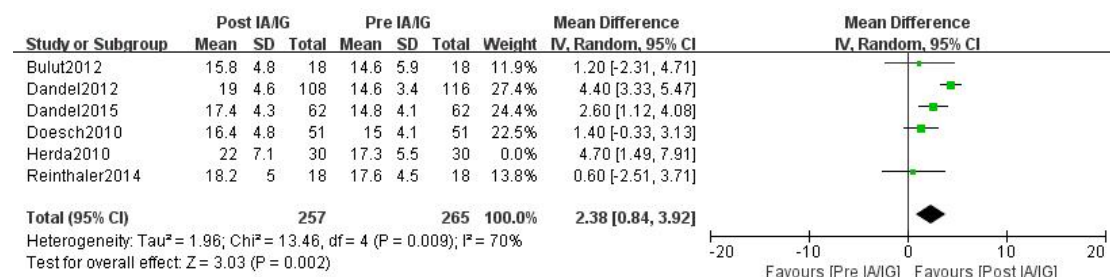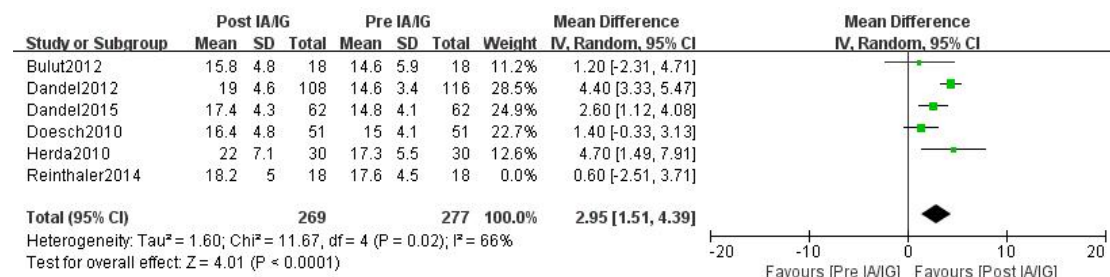

Supplement: Supplementary file 3 [file Datasheet2.pdf]
